# CD38 is methylated in prostate cancer and regulates extracellular NAD^+^

**DOI:** 10.1186/s40170-018-0186-3

**Published:** 2018-09-21

**Authors:** Jack Mottahedeh, Michael C. Haffner, Tristan R. Grogan, Takao Hashimoto, Preston D. Crowell, Himisha Beltran, Andrea Sboner, Rohan Bareja, David Esopi, William B. Isaacs, Srinivasan Yegnasubramanian, Matthew B. Rettig, David A. Elashoff, Elizabeth A. Platz, Angelo M. De Marzo, Michael A. Teitell, Andrew S. Goldstein

**Affiliations:** 1 Department of Molecular, Cell & Developmental Biology, University of California Los Angeles, Los Angeles, CA USA; 2 Sidney Kimmel Comprehensive Cancer Center at Johns Hopkins, Baltimore, MD USA; 3 Department of Pathology, Johns Hopkins University School of Medicine, Baltimore, MD USA; 4 Department of Medicine Statistics Core, University of California Los Angeles, Los Angeles, CA USA; 5 Molecular Biology Interdepartmental Program, University of California Los Angeles, Los Angeles, CA USA; 6 Department of Medicine, Division of Medical Oncology, Weill Cornell Medicine, New York, NY USA; 7 Englander Institute for Precision Medicine, Weill Cornell Medicine, New York, NY USA; 8 Department of Pathology and Laboratory Medicine, Weill Cornell Medicine, New York, NY USA; 9 Institute for Computational Biomedicine, Weill Cornell Medicine, New York, NY USA; 10 Department of Physiology and Biophysics, Weill Cornell Medicine, New York, NY USA; 11 James Buchanan Brady Urological Institute, School of Medicine, Johns Hopkins University, Baltimore, MD USA; 12 Departments of Oncology, Pathology, and Radiation Oncology and Molecular Radiation Sciences, Johns Hopkins University School of Medicine, Baltimore, MD USA; 13 Division of Hematology-Oncology, Department of Medicine, David Geffen School of Medicine, University of California Los Angeles, Los Angeles, CA USA; 14 Department of Urology, David Geffen School of Medicine, University of California Los Angeles, Los Angeles, CA USA; 15 Jonsson Comprehensive Cancer Center, University of California Los Angeles, Los Angeles, CA USA; 16 Veterans Administration Greater Los Angeles Healthcare System, Los Angeles, CA USA; 17 Department of Epidemiology, Johns Hopkins Bloomberg School of Public Health, Baltimore, MD USA; 18 Department of Oncology, Johns Hopkins University School of Medicine, Baltimore, MD USA; 19 Department of Urology and the James Buchanan Brady Urological Institute, School of Medicine, Johns Hopkins University, Baltimore, MD USA; 20 Department of Pathology & Laboratory Medicine, David Geffen School of Medicine, University of California Los Angeles, Los Angeles, CA USA; 21 Broad Stem Cell Research Center, University of California Los Angeles, Los Angeles, CA USA; 22 Molecular Biology Institute, University of California Los Angeles, Los Angeles, CA USA

**Keywords:** Prostate, CD38, NAD^+^, Methylation

## Abstract

**Background:**

Cancer cell metabolism requires sustained pools of intracellular nicotinamide adenine dinucleotide (NAD^+^) which is maintained by a balance of NAD^+^ hydrolase activity and NAD^+^ salvage activity. We recently reported that human prostate cancer can be initiated following oncogene expression in progenitor-like luminal cells marked by low expression of the NAD^+^-consuming enzyme CD38. CD38 expression is reduced in prostate cancer compared to benign prostate, suggesting that tumor cells may reduce CD38 expression in order to enhance pools of NAD^+^. However, little is known about how CD38 expression is repressed in advanced prostate cancer and whether CD38 plays a role in regulating NAD^+^ levels in prostate epithelial cells.

**Methods:**

CD38 expression, its association with recurrence after prostatectomy for clinically localized prostate cancer, and DNA methylation of the CD38 promoter were evaluated in human prostate tissues representing various stages of disease progression. CD38 was inducibly over-expressed in benign and malignant human prostate cell lines in order to determine the effects on cell proliferation and levels of NAD^+^ and NADH. NAD^+^ and NADH were also measured in urogenital tissues from wild-type and CD38 knockout mice.

**Results:**

CD38 mRNA expression was reduced in metastatic castration-resistant prostate cancer compared to localized prostate cancer. In a large cohort of men undergoing radical prostatectomy, CD38 protein expression was inversely correlated with recurrence. We identified methylation of the CD38 promoter in primary and metastatic prostate cancer. Over-expression of wild-type CD38, but not an NAD^+^ hydrolase-deficient mutant, depleted extracellular NAD^+^ levels in benign and malignant prostate cell lines. However, expression of CD38 did not significantly alter intracellular NAD^+^ levels in human prostate cell lines grown in vitro and in urogenital tissues isolated from wild-type and CD38 knockout mice.

**Conclusions:**

CD38 protein expression in prostate cancer is associated with risk of recurrence. Methylation results suggest that CD38 is epigenetically regulated in localized and metastatic prostate cancer tissues. Our study provides support for CD38 as a regulator of extracellular, but not intracellular, NAD^+^ in epithelial cells. These findings suggest that repression of CD38 by methylation may serve to increase the availability of extracellular NAD^+^ in prostate cancer tissues.

**Electronic supplementary material:**

The online version of this article (10.1186/s40170-018-0186-3) contains supplementary material, which is available to authorized users.

## Background

More than 164,000 men in the USA are estimated to be newly diagnosed with prostate cancer in 2018 and greater than 29,000 men in the USA will die of prostate cancer this year [1]. While the 5-year survival rate of patients with low-grade localized prostate cancer is high, men with metastatic prostate cancer suffer from a 30% 5-year survival rate, as tumors become increasingly difficult to treat [1]. Most patients with advanced metastatic prostate cancer are treated with therapies targeting the androgen signaling axis, which can be initially effective but tumors eventually recur in a lethal castration-resistant state [2]. A key reason why advanced prostate cancer is so difficult to treat is that disease progression and treatment resistance is associated with epithelial plasticity [3, 4], loss of differentiated luminal epithelial features [5], and gain of a stem/progenitor-like transcriptional program [6–8]. Changes in gene expression are also associated with a gain of functional progenitor capacity in treatment-resistant disease [9, 10].

We have isolated basal [11, 12] and luminal progenitor [7] cells from human prostate epithelium and demonstrated that tumors initiate in progenitor cells following oncogene expression, using an in vivo tissue regeneration and human prostate transformation assay [13]. We found that CD38 could separate progenitor-enriched (CD38^lo^) from differentiated (CD38^hi^) luminal cells in the human prostate [7] based on differences in colony-forming [14], sphere-forming [12], and organoid-forming [15] capacity. The CD38^hi^ cells expressed high levels of luminal differentiation markers including androgen receptor (AR) and AR target genes, while the CD38^lo^ cells showed evidence of NFkB signaling and expression of the anti-apoptotic factor BCL2 [7]. Following oncogene expression and transplantation, the CD38^lo^ luminal progenitors could give rise to highly proliferative tumors [7]. Given that basal cells are also CD38^lo^, we identified an inverse correlation between CD38 expression and progenitor activity.

CD38 is a glycoprotein that regulates cellular nicotinamide adenine dinucleotide (NAD^+^) metabolism through the hydrolysis of NAD^+^ into nicotinamide and cyclic ADP Ribose (cADPR) [16, 17]. CD38 is linked to calcium signaling through the production of cADPR [18]. CD38 localization has been reported both on the cell surface and in intracellular compartments [18, 19]. Increased expression of CD38 is found in diverse hematological malignancies where it serves as a therapeutic target on the cell surface [20–23]. However, little is known about CD38 function in epithelial tissues. Deletion of CD38 in kidney epithelial cells initiates an epithelial-mesenchymal transition with loss of epithelial markers P-cadherin and ZO-1 and gain of mesenchymal markers desmin and α-smooth muscle actin [24]. In a limited cohort, a few studies have reported loss of CD38 expression in prostate cancer [25–27]. We have confirmed and extended these findings on a larger cohort of tissue samples, demonstrating that CD38 expression is inversely correlated with disease pathology, from benign prostate tissue to prostatic intraepithelial neoplasia (PIN) to low-grade cancer to high-grade cancer [7]. Using a global CD38 knockout mouse, Aksoy et al. reported significantly elevated NAD^+^ levels in multiple tissues including brain, heart, lung, and liver extracts compared to WT animals [28]. As mice age, NAD^+^ levels decline in several tissues which is associated with a decline in mitochondrial and adult stem cell function [29]. Interestingly, CD38 knockout mice are protected from an age-related decline in NAD^+^ levels [20]. These data suggest that loss of CD38 in prostate cancer may contribute to elevated NAD^+^ levels in tumor tissues.

NAD^+^ is a metabolic co-factor and electron carrier that is critically important for both glycolysis and oxidative phosphorylation, as well as for the NAD^+^-dependent deacetylases in the sirtuin family [30]. In order to supply sufficient pools of this metabolic co-factor, NAD^+^ is generated from precursors including tryptophan, nicotinic acid, nicotinamide, and nicotinamide riboside (NR) through multiple pathways [31]. In the salvage pathway, nicotinamide phosphoribosyl-transferase (NAMPT) converts nicotinamide into nicotinamide mononucleotide (NMN) which can be further processed by nicotinamide mononucleotide adenylyltransferase (NMNAT) into NAD^+^. Expression of NAMPT is elevated in prostate cancer tissues, and inhibition of NAMPT, through shRNA-mediated knockdown or small molecules, impairs proliferation of prostate cancer cell lines [32]. While intracellular NAD^+^ levels maintain vital functions for epithelial cells, extracellular NAD^+^ may function in immune suppression. Addition of NAD^+^, but not nicotinamide or cADPR, has been shown to inhibit T cell proliferation, cytotoxic activity, and survival through the P2X7 receptor [33, 34]. This process has been termed NAD^+^-induced cell death [35] and has been observed in multiple T cell subsets [33, 36]. Modulation of the immune microenvironment can have a significant impact on tumor progression and metastasis [37]. Several key questions remain unanswered, including (1) What is the level of CD38 expression in metastatic castration-resistant prostate cancer? (2) What mechanisms are responsible for low expression of CD38 in primary and metastatic prostate cancer? and (3) What are the functional consequences of CD38 expression in benign and malignant prostate epithelial cells? In this study, we address these questions using a range of approaches to evaluate prostate cancer tissues and cell lines.

## Methods

### Methylation analysis

Locus-specific DNA methylation analyses were performed as described by Yegnasubramanian et al. with minor modifications [38]. Patient samples used in this study were described previously [39, 40]. In brief, DNA sample concentrations were determined by a spectrophotometer and validated by quantitative real-time PCR. DNA samples were digested with AluI and HhaI (New England Biolabs). Enrichment reactions containing 20 μl of magnetic Tylon beads (Clontech), 10 μg of recombinant MBD2-MBD (Clontech), and 200 ng of unmethylated self-ligated pCR2.1 vector (Invitrogen) were assembled in enrichment buffer (4% glycerol, 1 mM MgCl_2_, 0.5 mM EDTA, 0.5 mM DTT, 120 mM NaCl, 10 mM Tris–HCl (pH 7.4), 0.2% Tween-20, and protease inhibitors) and incubated for 1 h at room temperature. After immobilization of magnetic beads, the supernatants containing unbound MBD2-MBD polypeptides were discarded and digested DNA samples were diluted in 100 μl of enrichment buffer and incubated with MBD2-MBD-coated beads for 12 h at room temperature under gentle shaking. Beads and associated DNA complexes were then immobilized by magnetization and washed three times with enrichment buffer. Precipitated DNA containing methylated DNA fragments were eluted in water by heating to 95 °C for 15 min. Eluted DNA was then subjected to quantitative real-time PCR using the IQ SYBR Green Supermix (Biorad) using CD38-F: ATCCTCGTCGTGGTGCTC and CD38-R: CTTAGTCGCCAACCCACCT primers. Male white blood cell (WBC) genomic DNA was in vitro treated with M.SssI (NEB) to obtain a fully methylated control. Untreated male WBC DNA served as a negative control. For quantitative assessment of locus-specific methylation levels, *C*_*t*_ values of the samples of interest were normalized to *C*_*t*_ values of the positive control (SssI) and calculated methylation indices (ranging from 0.0 to 1.0) were used to derive methylation heatmaps. For in silico analysis of TCGA data correlating mRNA expression (RNA-seq, RSEM z-scores) and methylation (Infinium Human Methylation 450k BeadChip analysis) in primary prostate cancer samples, data were obtained from Cancer Genome Atlas Research Network [41] and were analyzed using cBioPortal [42]. Prostate cancer cell lines CWR22rv1, DU145, LNCaP, and LAPC4 were treated with 100 nM, 500 nM decitabine, or solvent (DMSO) for 4 days. Expression of *CD38* was determined by quantitative real-time PCR using primers:

- CD38-F GCTCAATGGATCCCGCAGTA
- CD38-R GGATCCTGGCATAAGTCTCTGG
- GAPDH-F CCATCAAGTCCACAACACGGTTGCTGTA
- GAPDH-R GTCTTATGACCACTGTCCATGCCATCAC

### Expression from RNA sequencing

RNA-seq data from the Weill Cornell Cohort included 164 samples: 31 benign prostate, 74 localized prostate cancer (PCa), and 41 CRPC-Adeno, and 18 CRPC-NE cases [43–45]. Tissue extraction from frozen material and RNA-seq processing and sequencing was performed according to the protocols previously described [43–45]. All reads were independently aligned with STAR_2.4.0f1 [46] for sequence alignment against the human genome build hg19, downloaded via the UCSC genome browser [http://hgdownload.soe.ucsc.edu/goldenPath/hg19/bigZips/], and SAMTOOLS v0.1.19 [47] for sorting and indexing reads. Cufflinks (2.0.2) [48] was used to quantify expression levels for all annotated genes in Gencode v19 [49] GTF annotation file. Expression levels are described by FPKM (fragment per kilobase of exonic regions per million mapped reads). Since the sequenced samples from the published cohorts were processed using different library preps, batch normalization was done using Combat from sva bioconductor package [50].

### Tissue microarrays

The PSA progression tissue microarray was obtained through the Prostate Cancer Biorepository Network (PCBN) and has been previously described [51, 52]. For the majority of patients included, four cancer cores and two benign cores are included, and we plotted the mean score for each man’s cancer or benign tissue cores. Immunohistochemical staining was performed as described [7] using antibodies against CD38 (LifeSpan BioSciences, LS-A9696). Staining scores were calculated by multiplying the percentage of CD38+ cells (from 0 to 100%) by the intensity of staining (from 0 to 3), as determined by a pathologist (M.A.T.). such that composite scores ranged from 0 to 300.

### Statistical methods

Patient characteristics were summarized using means/SD and frequencies/percentages and then formally compared between recurrence/non-recurrence patients using paired *t* tests or McNemar’s tests as appropriate (Table 1). The distribution of the average CD38 scores between cancer/benign biopsy cores were compared using a Wilcoxon test. The association between CD38 and recurrence was assessed using conditional logistic regression, taking into account the matching factors [53] (age, race, Gleason, pathologic stage) and adjusting for pre-operative PSA and surgical margin status. Three models were run, each with the covariates mentioned above and CD38 as above the median (1.25) in cancer cores, above the median (195) in benign cores, and above the median in both benign and cancer cores. For our validation data (UCLA-TMA [54]), we computed the same groups (above/below median using cancer median of 36.7 and benign median of 160) and constructed Kaplan Meier curves to assess recurrence-free survival between groups using the Breslow test. Statistical analyses were run using SAS 9.2 (SAS institute, Cary, NC, USA). Relative NAD^+^/protein or NAD^+^/DNA levels were compared between groups using the two-sample *t* test. All statistical tests were two-tailed. *p < 0.05, **p < 0.005, ***p < 0.0005, and ****p < 0.0001 were computed using GraphPad Prism software.

**Table 1:** Patient characteristics in PSA progression tissue microarray

Characteristic	No recurrence (*n* = 499)	Recurrence (*n* = 499)	*p* value
Age at surgery	59.0 (6.0)	58.8 (6.3)	Matched
Race			Matched
AA	39 (7.8%)	47 (9.4%)	
White	440 (88.2%)	426 (85.4%)	
Other	11 (2.2%)	19 (3.8%)	
Gleason sum			Matched
5	3 (0.6%)	3 (0.6%)	
6	69 (13.8%)	68 (13.6%)	
7	314 (62.9%)	305 (61.1%)	
8	113 (22.6%)	123 (24.6%)	
S stage			Matched
1	67 (13.4%)	66 (13.2%)	
2	260 (52.1%)	260 (52.1%)	
3	172 (34.5%)	173 (34.7%)	
Pre-op PSA	11.0 (8.3)	12.3 (10.2)	0.026
Surgical margins +	110 (22.0%)	176 (35.3%)	< 0.001

The PSA progression tissue microarray contains tissue cores from 499 men who developed biochemical recurrence and 499 controls who did not develop recurrence, matched based on age, race, Gleason sum, and pathologic stage. Patient characteristics were summarized using means/standard deviation and frequencies/percentages

### Engineering expression of CD38

Using MGC Human CD38 sequence-verified cDNA (Clone ID: 4309086, Dharmacon) as a template, we amplified full-length CD38 with forward primer (GTACAGCGCTGAGTTCGAACCATGGCCAACTGCGA) and reverse primer (GTACAGCGCTCTCGAGCTAGATCTCAGATGTGCA) and cloned into the inducible pSTV lentiviral vector using the AFE1 restriction site and Afe1 (New England Biolabs). The E226Q point mutant was generated by using QuickChange Lightning Site-Directed Mutagenesis Kit (Agilent) according to manufacturer’s protocol with forward primer (GTTGCAAATTATGGACTTGCACACTCCCAAAAGTGCT) and reverse primer (AGCACTTTTGGGAGTGTGCAAGTCCATAATTTGCAAC). CD38 expression is under the control of the Tet Response Element, which allows gene induction following addition of doxycycline (Dox, Sigma). In the same vector, GFP is constitutively expressed under the human ubiquitin promoter, allowing for isolation of highly transduced cells by fluorescence-activated cell sorting.

### Cell lines, lentiviral transduction, and proliferation assays

Cell lines used in this study (RWPE1, LNCaP, DU145, PC3) were purchased from the ATCC and cultured in the recommended media. Cells were grown on plates coated with 0.01% poly-l-Lysine (Sigma) to enhance attachment. Cell lines were transduced at high MOI, expanded for 4–7 days of growth, and sorted for GFP expression using a BD FACS ARIA II prior to initial experiments. Cultures were expanded and maintain in the absence of Dox unless otherwise indicated. To measure cellular growth, cells were plated onto black wall clear bottom tissue culture plates. One hundred microliters of cells diluted at final concentration of 20,000–60,000/ml under different experimental conditions was plated. At different time points, 11 μl of 0.5 mg/ml of Resazurin-Na salt (alamarBlue) diluted in media was added to wells of interest and plates were continued to be incubated at 37 °C. Exactly 1 h later, total fluorescence was measured using a Tecan plate reader (Infinite M1000) with 555 ± 7 nm excitation and 590 ± 10 nm emission. Four different spots/well were measured, and third highest value was used for analysis. To measure cellular growth by increase in total DNA content, the media containing Resazurin was removed and cells were lysed by addition of 50 μl of buffer (0.1% SDS, 20 mM NaOH, and 1 mM EDTA) and returned to 37 °C incubator for additional data points. A day after the last data point was collected, 100 μl of buffer (30 mM Tris-HCl pH 7.4 and 6 μg/ml of Hoechst-33342) is added to wells, gently mixed by tapping the plate and incubated at 37 °C overnight. The next day, plates were given time to come to room temperature and analyzed using plate reader with 355 ± 10 nm excitation and 465 ± 10 nm emission.

### Western blot

Cells grown in the absence of doxycycline were plated with 0 or 20 ng/ml of Dox added at the time of seeding for a total of 4 days. To collect protein, media was removed and cells were washed with HBSS and lysed in 300 μl of RIPA buffer and protease inhibitor (Roche). Cells were scraped from the plate, moved to Eppendorf tubes, and spun at 17,000×*g* for 10 min prior to storing the samples at − 20 °C. Total protein was determined using the BCA assay and ~ 5 μg of protein was separated on a 4–12% Bis-Tris PAGE. Proteins were transferred onto PVDF membranes and probed with primary antibodies followed by HRP conjugated secondary antibodies and detected with HRP chemiluminescence. Primary antibodies used were CD38 (Santa Cruz sc-374650), Alpha-tubulin (Developmental Studies Hybridoma Bank #12G10), NAMPT (Cell Signaling D7V5J), and NAPRT (Proteintech 13549-1-AP).

### NAD^+^/ NADH measurement from cell cultures

Cells were cultured in 24-well plates. Prior to NAD^+^/NADH measurement, media was removed and cells were washed with 500 μl of HBSS. Shortly after HBSS removal, 200 μl of 1:1 mixture of 0.4% SDS + 80 mM NaOH:PBS is added. After 10–15 min incubation at room temperature, the pH is adjusted by adding 400 μl of 18 mM HCl. DNA from cells was sheered by passing through a 25-G needle. 60 μl of the homogenate was transferred into mini-PCR tubes; we added 15 μl of 0.5 M HCl (for NAD^+^) or NaOH (for NADH) and tubes were incubated at 60 °C for 15 min. Tubes were cooled and 50 μl of buffer (300 mM Tris-base +160mMNaOH or 300 mM Tris-base +160 mM HCl) was added for NAD^+^ or NADH respectively. NAD^+^/NADH was measured independently using NAD^+^/NADH-Glo Assay (Promega) and normalized based on total protein and/or DNA. Protein concentrations were determined using 20 μl of homogenate with BCA assay. DNA concentrations were determined by mixing 60 μl of homogenate with 60 μl of 20 mM Tris-HCl, pH 7.4 and 10 μg/ml of Hoechst-33342, in Clear Bottom Black Polystyrene Microplates, against a DNA standard diluted in the same buffer. DNA to protein ratio was used to evaluate sample quality, purity, and well to well variation. Cellular permeabilization was achieved by addition of 100 μl of HBSS + 0.5% Triton X-100 after removal of HBSS wash solution. Plates are incubated for 15 min at room temperature prior to adding 200 μl of 1:1 mixture described above (0.4% SDS + 80 mM NaOH:PBS). Control wells received 200 μl of 1:1 mixture first, and after 15 min, 100 μl of HBSS +Triton X-100 was added. All wells were neutralized by addition of 300 μl of 25 mM HCl.

### NAMPT inhibition with FK866

To see the effect of NAMPT inhibition on growth rate or NAD^+^/NADH levels, cells were seeded in the presence of FK866 (Sigma) diluted in 100% EtOH and added to media to reach the concentration indicated. Media was changed with fresh drug every 48 h. Changes in total DNA measurements were used for growth rate analysis. NAD^+^/NADH was measured on day 3.

### Measurement of extracellular NAD^+^ hydrolase activity

To test for extracellular NAD^+^ hydrolase activity, transduced cells were seeded onto 24-well plates ± 20 ng/ml of Dox for a total of 4 days. Media was replaced with fresh media ± Dox 24 h prior to evaluation of NAD^+^ hydrolase activity. Wells were washed with HBSS, and 200 μl of HBSS + 800 nM NAD^+^ (Sigma, diluted in PBS) was added to each well and incubated at room temperature for 30 min. Media was removed from wells, centrifuged at 800×*g* for 10 min, and 60 μl was moved into a new tube and treated to measure NAD^+^ by addition of 15 μl of 0.5 M HCl, heating, and neutralization. A serial dilution of starting material was also generated and treated under similar conditions as a standard. To measure the effect of extracellular NAD^+^ on the internal pool: supernatant was removed, cells were washed with 1 ml of HBSS, and stopped by addition of 200 μl of 1:1 mixture (0.4% SDS + 80 mM NaOH:PBS) followed by HCl neutralization.

### NAD^+^/NADH measurements in mouse tissues

C57BL/6J (WT) and B6.129P2-Cd38tm1Lnd/J (CD38KO) mice were obtained from Jackson Labs and housed and bred under the care of the Division of Laboratory and Animal Medicine (DLAM) at UCLA according to approved protocols. To measure total NAD^+^/NADH from prostate, seminal vesicles, and liver, tissues were obtained from adult mice (aged 8–16 weeks), rinsed with PBS, and stored in 0.8 ml PBS on ice. 0.8 ml of 0.4% SDS + 80 mM NaOH was added to each sample, and it was immediately sonicated on ice for 20–25 bursts (20-s pulses separated by 10 s) allowing complete dissociation of the tissue. Samples were maintained at room temperature to minimize additional precipitation. To study the contribution of seminal vesicle tissue without the fluid, seminal vesicles were cut on the coronal plane and fluid was pushed out. Remaining seminal vesicle tissues were rinsed with HBSS, placed into PBS on ice, and homogenized as described for total tissue. For DNA normalization, 200 μl of the sonicated homogenate was mixed with equal volume of 100 mM NaCl, 40 mM HCl, 20 mM Tris-HCl (pH 7.4), and 10 mM EDTA. 2.5 μl of Proteinase K (20 mg/ml, Thermo Scientific) was added to each tube, and the mixture was incubated at 55 °C for 2–3 h with shaking. Samples were allowed to come to room temperature and DNA was extracted with 500 μl of chloroform. Fifty microliters of the extracted samples is mixed with equal volume of 10 μg/ml of Hoechst (Invitrogen) for DNA measurement against a known DNA standard.

### Isolation of total mouse prostate and seminal vesicle cells for flow cytometry and NAD^+^ hydrolase activity

Total cells from prostate were obtained by modification of our published work [14]. The urogenital system was collected and placed in base media of RPMI (Gibco) + 10% FBS (Corning). Isolated prostates were moved onto a clean surface and minced using a razor blade. Tissue pieces were enzymatically digested in 1 mg/ml of Dispase (Gibco) and 1 mg/ml of collagenase type I (Gibco) diluted in base media and incubated at 37 °C on an orbital rocker for 60–90 min. Digested tissues are centrifuged at 800×*g* for 5 min and washed with PBS (Gibco). After 800×*g* centrifugation, PBS is removed, 500 μl of warm TryplE (Gibco) is added, and tubes are mixed and incubated at 37 °C water bath for 15 min. TryplE is inactivated by addition of 1.3 ml of base media. DNase I (Sigma) is added at a final concentration of 100 μg/ml, and tubes are incubated for an additional 10 min. The tissue is further dissociated mechanically using P-1000 pipet tips and passed through a 70-μm cell strainer. For flow cytometry, cells were stained with antibodies against mouse EpCAM-APC (clone G8.8, eBiosciences) and CD38-PE (clone 90, BioLegend) and evaluated using FACSDIVA software on a FACSCanto cytometer (BD Biosciences). To further purify viable seminal vesicle cells, 1× Percoll solution was made by adding 1 ml of 11× buffer (1100 mM Glycerol, 880 mM NaCl, 220 mM HEPES, and 4.4 mg/ml BSA) to 10 ml of Percoll (GE Life Sciences). After preparing a 1:1 mixture of cells suspended in 0.5 ml base media with 1× Percoll solution, samples are centrifuged at 800×*g* for 5 min. The supernatant containing cells floating at the top is moved to a new tube and is mixed at a 2:1 ratio with 4× buffer (4 ml of 11× buffer and 7 ml of RPMI + 10% FBS). The mixture is centrifuged at 3000×*g* for 5 min, and the supernatant is discarded. The pellet containing nearly 90–100% live cells is gradually increased in volume with base media. Cells suspend in base media are incubated at 37 °C for 30–40 min prior to analysis. To measure NAD^+^ hydrolase activity of total cells, Percoll-purified seminal vesicle cells were centrifuged at 800×*g* for 5 min and washed once with HBSS. Using a 0.2-ml PCR tube, each sample (~ 500,000) was suspended in 50 μl of HBSS to which 50 μl of 2 nM of NAD^+^-HBSS or HBSS was added. Samples were incubated in a 37 °C water bath for 50–60 min, with gentle mixing every 15 min. Cells were centrifuged at 800×*g* for 10 min, and 60 μl of the supernatant was used for NAD^+^ measurement. To measure intracellular NAD^+^, additional 20 μl of supernatant was removed from each tube and cells were washed with 180 μl of HBSS. After pelleting cells at 800×*g* for 10 min, most of the solution is removed and cells are suspended in 50 μl of HBSS followed by addition of 50 μl of 0.4% SDS and 200 mM HCl.

### Collection of blood and purified liver cells from mice

Mice were euthanized by CO_2_ inhalation and checked for the absence of response to pressure stimulus. Next, the chest cavity was exposed and 0.3–0.5 ml of blood was collected from the aorta using a 22-G needle and syringe pre-coated with (20 mg/ml) heparin. The blood was immediately moved to 0.5-ml tubes and centrifuged at 3000×*g* for 5 min in 4 °C. The supernatant was moved to a new tube and was further centrifuged at 17,000×*g* for 5 min. Finally, 40 μl of serum in duplicate was added to 10 μl of 0.5 M HCl, mixed, and stored on ice prior to starting the next animal. All animals used were 10–12 weeks of age. NAD^+^ was measured as described above, comparing wild-type and CD38 knockout mice. After collecting blood for NAD^+^ measurement, mice were perfused with 10 ml of HBSS. Liver tissue was moved to base media prior to dissociation. After mincing the liver, it was further digested in 5 ml of base media containing 0.5 mg/ml of Dispase and 0.5 mg/ml collagenase for 30–40 min at 37 °C on an orbital rocker. Partially dissociated tissues were pelleted, washed with PBS, digested with TryplE, neutralized, treated with DNase, diluted, and passed through 70-μM cell strainers. After centrifuging cells at 1000×*g*, the pellet was suspended in 1 ml of base media and was used for further Percoll purification, similar to seminal vesicle tissue described above. The final purified cell preparations contained less than 10% red blood cells. Percoll-purified seminal vesicle and liver cells were subjected to lysis and western blotting to analyze CD38 expression.

## Results

### *CD38* mRNA is reduced in metastatic castration-resistant prostate cancer

**Fig. 1:**
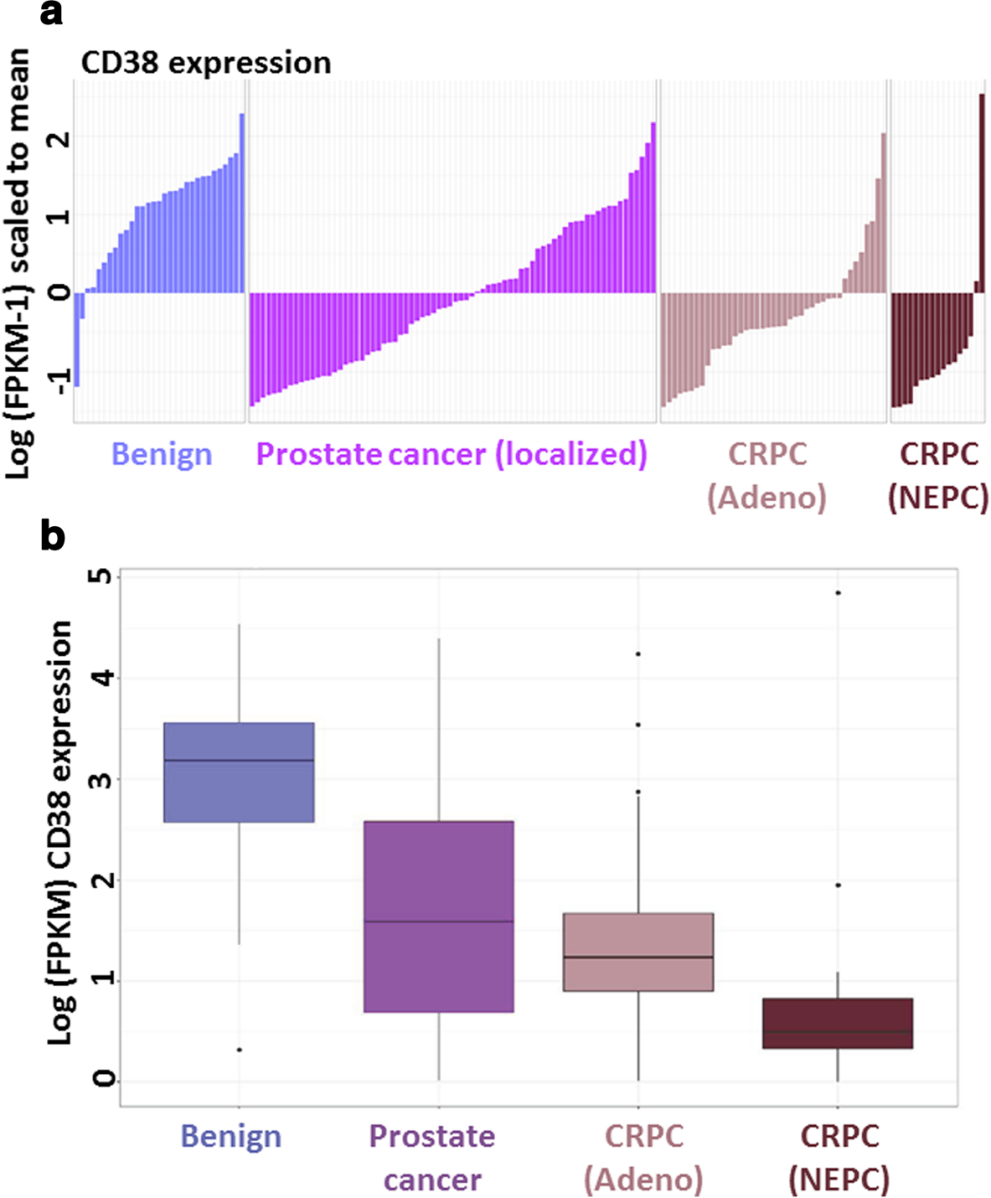
Reduced expression of *CD38* in metastatic prostate cancer. **a** Waterfall plot indicating relative *CD38* mRNA expression from RNA sequencing of benign prostate, localized prostate cancer, metastatic castration-resistant prostate cancer (CRPC) with an adenocarcinoma (adeno) phenotype and metastatic CRPC with a neuroendocrine prostate cancer (NEPC) phenotype. Expression levels are presented as Log of FPKM-1 (Fragments Per Kilobase Million) values and scaled to the mean of all values shown. **b** Box plot corresponding to samples from **a** presented as Log of FPKM values

We previously demonstrated that *CD38* mRNA and protein expression is reduced in primary prostate cancer [7], but the expression of *CD38* in metastatic prostate cancer is poorly defined. When comparing gene expression of metastatic prostate tumor samples recovered from autopsies to normal prostate, we identified *CD38* as significantly downregulated in metastases [55]. To further evaluate *CD38* expression in advanced disease, we interrogated transcriptional profiles of patient tissues representing benign prostate, primary prostate cancer, and castration-resistant prostate cancer (CRPC). The CRPC samples were subdivided into adenocarcinoma and neuroendocrine prostate cancer (NEPC) based on histological and molecular features [45]. RNA sequencing data indicates a clear trend with *CD38* mRNA expression lower in primary prostate cancer than in benign prostate, and further reduced in CRPC tissues (Fig. 1). Within CRPC subsets, *CD38* mRNA expression is lowest in NEPC tissues.

### High expression of CD38 in prostate cancer is associated with reduced risk for recurrence

Based on low expression of *CD38* in CRPC samples, we hypothesized that CD38 protein expression in primary prostate cancer may be predictive of disease progression. We previously reported that low *CD38* mRNA expression in radical prostatectomy tissues is prognostic for biochemical recurrence and metastasis [7]. A recent study determined that combined expression of CD38 and ARG2 was prognostic for recurrence-free survival in a Stanford tissue cohort, while low expression of CD38 was associated with recurrence-free survival in the Canary tissue cohort [56]. We evaluated CD38 expression in the PSA progression tissue microarray (TMA) [51, 52] which contains prostatectomy tissues from 499 patients who developed biochemical recurrence as well as 499 control patients who did not develop recurrence, matched on age, race, pathological stage (T2, T3a, T3b, etc.), and Gleason sum (Table 1).

**Fig. 2:**
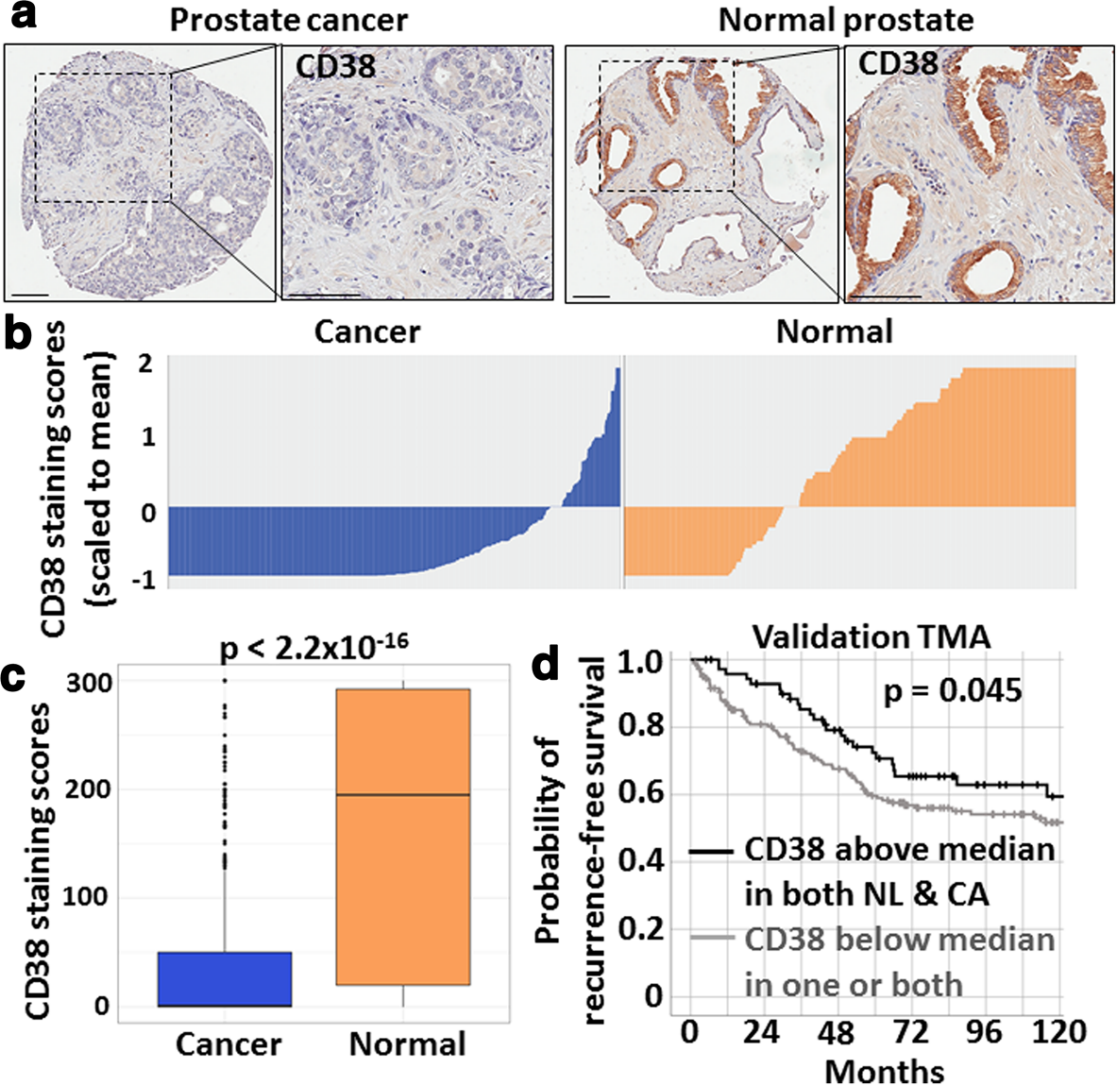
Association of CD38 protein expression with prostate cancer recurrence. **a** Representative images of CD38 staining in normal and cancer cores from the PSA progression tissue microarray. Scale bars represent 100 μm. **b** Waterfall plot representing composite CD38 staining scores in normal and cancer cores scaled to the mean of all values. **c** Box plot of composite CD38 staining scores. Statistics represent Welch Two Sample *t* test. **d** Kaplan-Meier plot of biochemical recurrence-free survival for patients with CD38 staining scores greater than the median in both normal and cancer cores compared to all other patients (remainder). Breslow test *p* value is shown, with a hazard ratio of 0.71

Overall, cancer tissues contained significantly lower CD38 staining scores than normal prostate (Fig. 2a–c). When cancer samples were split into two groups defined as above or below the median CD38 staining score, we found that higher CD38 (above the median) in cancer specimens was statistically significantly inversely associated with risk of recurrence (OR = 0.76, 95% CI 0.59–0.98, *p* = 0.031) (Table 2). These data are consistent with mRNA expression [7] and the Stanford/Canary tissue cohorts [56], indicating that low CD38 is associated with risk of recurrence. Gleason sum was not found to be a significant predictor of recurrence (Table 2) in this TMA, likely because the recurrence and no recurrence groups were well matched based on Gleason sum (Table 1).

**Table 2:** Higher CD38 is associated with a lower risk of recurrence after prostatectomy for clinically localized disease

Characteristic	Model 1	*p* value	Model 2	*p* value	Model 3	*p* value
	Odds ratio (95% CI)		Odds ratio (95% CI)		Odds ratio (95% CI)	
Age at surgery (years)	0.81 (0.70–0.93)	0.003	0.81 (0.71–0.93)	0.003	0.81 (0.71–0.93)	0.003
Caucasian (yes/no)	0.09 (0.02–0.41)	0.002	0.10 (0.02–0.45)	0.003	0.11 (0.02–0.49)	0.004
High Gleason sum (yes/no)	1.44 (0.08–27.4)	0.819	1.80 (0.10–34.1)	0.715	1.41 (0.07–26.7)	0.831
S stage (yes/no)	2.09 (0.26–16.59)	0.487	2.06 (0.25–17.00)	0.502	2.31 (0.25–21.07)	0.457
Pre-op PSA (1 unit)	1.01 (0.99–1.03)	0.227	1.01 (0.99–1.03)	0.282	1.01 (0.99–1.03)	0.274
Surgical margins (+/−)	2.33 (1.65–3.30)	< 0.001	2.25 (1.60–3.17)	< 0.001	2.37 (1.68–3.36)	< 0.001
CD38 above med. cancer	0.69 (0.52–0.91)	0.007	XX	XX	XX	XX
CD38 above med. benign	XX	XX	0.91 (0.69–1.20)	0.505	XX	XX
CD38 above med. for both	XX	XX	XX	XX	0.65 (0.48–0.88)	0.006

Three conditional logistic regression models were run, each with the covariates mentioned above and CD38 as above the median in cancer cores (model 1), above the median in benign cores (model 2), and above the median in both (model 3). Odds ratios and 95% confidence intervals were extracted from each model

When we analyzed the study looking only at normal (cancer-adjacent) glands, we did not find a statistically significant association between CD38 staining scores and recurrence (Table 2). However, when combining CD38 scores in normal and cancer, we found that high expression of CD38 (above the median) in both normal and cancer tissues is significantly associated with a reduced risk of recurrence (*p* = 0.018) when compared to all other combinations (above median cancer and below median normal, below median cancer and above median normal, below median cancer and below median normal). We found that high expression of CD38 in normal and cancer tissues is associated with a 21% lower risk of recurrence (OR = 0.79, 95% CI 0.63–0.98, *p* = 0.037) compared to patients who do not have high CD38 in both. We analyzed a separate TMA [54] for CD38 expression and observed a similar pattern, with high (above the median) expression for CD38 in both benign and cancer tissues associated with reduced risk of recurrence (Fig. 2d).

### Evidence for epigenetic regulation of CD38 in prostate cancer

**Fig. 3:**
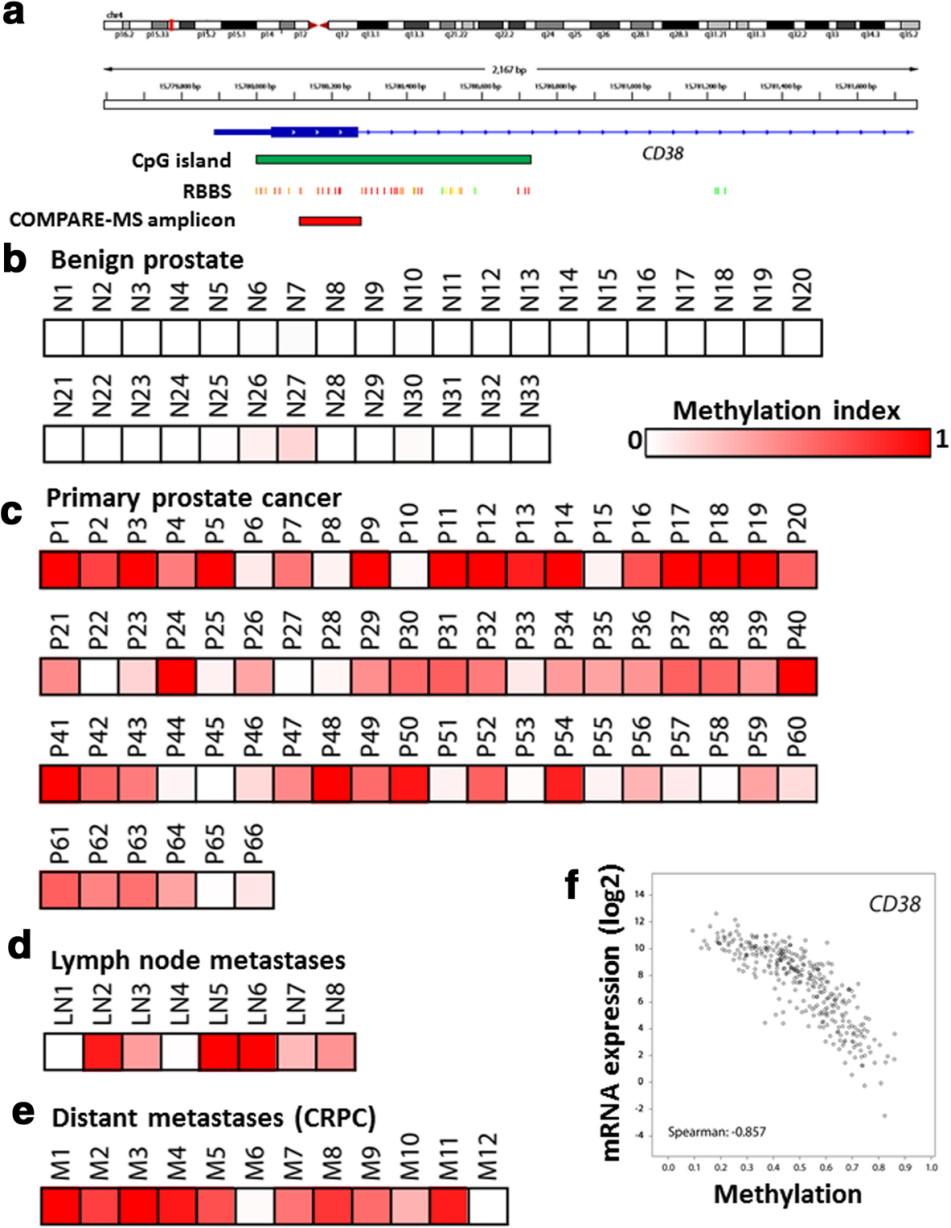
The *CD38* locus undergoes CpG hypermethylation in prostate cancer. **a** Schematic representation of the *CD38* locus. Note that the CpG island (green box) extends downstream of the transcriptional start site into the first intron. In silico analysis of publicly available reduced representation bisulfite sequencing (RRBS) is shown below (heat map: red—dense methylation, green—no methylation). The location of the PCR amplicon used in subsequent COMPARE-MS experiments is indicated by the red box. **b**–**e** Methylation heat maps derived from COMPARE-MS analysis of benign prostate tissue (**b**), primary prostate cancers (**c**), and prostate cancer metastases (**d**, **e**) show hypermethylation of the CD38 in primary prostate cancer and prostate cancer metastases (heat map: red—dense methylation; white—no methylation). **f** In silico analysis of TCGA data reveals frequent hypermethylation of the *CD38* locus. Correlation plots of log2 mRNA expression (based on RNA-seq, RSEM z-scores) and methylation levels (based on Infinium Human Methylation 450k BeadChip analysis) in 333 primary prostate cancer samples

To test if *CD38* is regulated epigenetically by CpG methylation, we first performed in silico analysis and identified a CpG island in close proximity to the transcriptional start site of *CD38*. Furthermore, re-analysis of publicly available reduced representation bisulfite sequencing (RRBS) data [57] for the prostate cancer cell line LNCaP demonstrated that dense CpG methylation was present around the transcriptional start site and in the first exon of *CD38* (Fig. 3a). To further investigate the methylation status of *CD38* in prostate cancer cell lines and clinical specimens, we used a previously described and extensively validated combined methylation-sensitive restriction enzyme digestion and methylated-DNA precipitation assay (COMPARE-MS) [38, 58, 59]. Whereas benign primary prostate epithelial cells (PrEC) and immortalized prostate epithelial cells (RWPE-1) showed no methylation, all prostate cancer cell lines (including LNCaP, LNCaP-abl, C4-2B, LAPC4, VCaP, DU145, PC3, and CWR22rv1) showed evidence of dense CpG methylation around the first exon of *CD38* (Additional file 1). Furthermore, analyses of clinical specimens showed low-level methylation in 2 of 33 benign prostate tissue samples; in contrast, 52 out of 66 primary carcinoma samples exhibited CpG methylation (Fig. 3b, c). Similarly, 6 out of 8 lymph node metastases and 10 out of 12 distant metastases showed methylation of *CD38* (Fig. 3d, e). These observations were further corroborated by in silico analyses of TCGA data [41] obtained using cBioPortal [42], which demonstrated high-level CpG methylation of the *CD38* locus, comparable to other known frequently methylated genes in prostate cancer such as *GSTP1* and *PTGS2* (Additional file 2). Importantly, these in silico analyses demonstrate a tight correlation between *CD38* methylation and mRNA expression (Fig. 3f). In addition, pharmacologic unmasking experiments using the DNA methyltransferase inhibitor 2′-deoxy-5-azacytidine (DAC) resulted in re-expression of *CD38* in a subset of cell lines with dense *CD38* CpG island methylation (Additional file 3), further supporting the notion that *CD38* expression is at least partially regulated by CpG methylation in prostate cancer. Taken together, these results suggest that the *CD38* locus undergoes hypermethylation changes that are present in primary tumors and maintained in regional and distant metastases.

### CD38 expression does not significantly alter cell proliferation or intracellular NAD^+^ levels

**Fig. 4:**
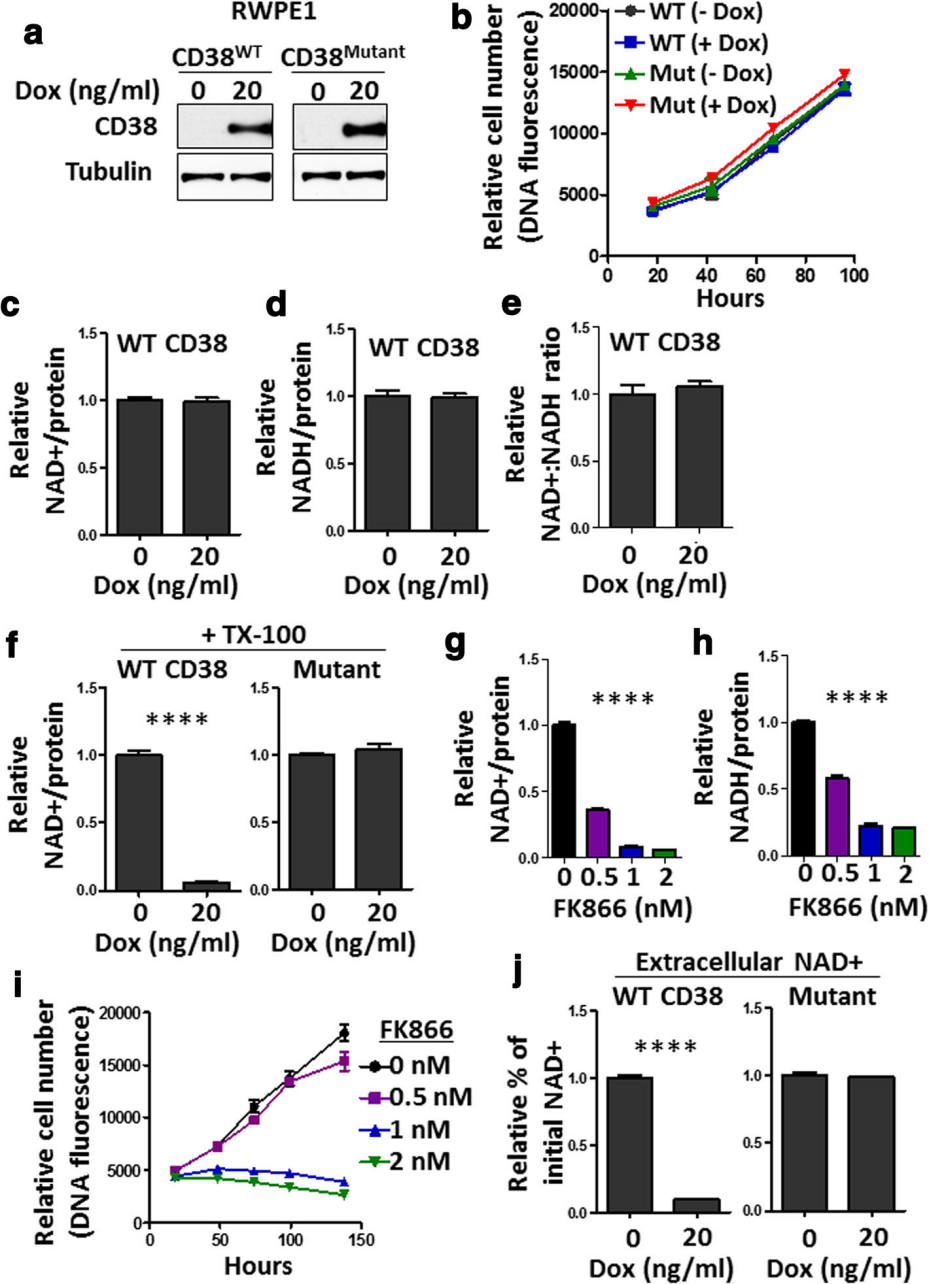
CD38 regulates extracellular but not intracellular NAD^+^ levels in RPWE1 cells. **a** Western blots demonstrate doxycyline (Dox) induced expression of wild-type (WT) or mutant (E226Q) CD38 in RWPE1 cells. Tubulin serves as a loading control. **b** Cell proliferation assay over 4 days in culture in the presence or absence of 20 ng/mL Dox. Relative cell number was assessed by measuring DNA fluorescence at 465 nm. 3–6 replicate wells per group per time point were measured. Plot shows mean ± standard error of the mean (SEM). **c**, **d** NAD^+^ and NADH levels were measured relative to total protein in each sample and presented relative to no Dox (non-induced) sample. Mean ± SEM of four replicates is shown. **e** NAD^+^:NADH ratio is calculated based on results shown in **c** and **d**. Mean ± SEM of four replicates is shown. **f** Cells were treated with Triton X-100 (TX-100) to permeabilize cells followed by NAD^+^ measurements. NAD^+^/protein is shown relative to no Dox. Mean ± SEM of four replicates is shown. **g**–**i** RWPE1 cells were treated with increasing concentrations of FK866 followed by NAD^+^ (**g**) and NADH (**h**) measurements. Mean ± SEM of four replicates is shown. Newman-Keuls Multiple Comparison Test. **i** Cell proliferation assay over 4 days in culture in the presence of the indicated concentrations of FK866. DNA fluorescence represents relative cell number. 3–6 replicate wells per group per time point were measured. Plot shows mean ± standard error of the mean (SEM). **j** Relative NAD^+^/protein levels in the media 30 min after the addition of 800 nM exogenous NAD^+^. Mean ± SEM of four replicates is shown

To assess the functional role of CD38 in prostate epithelial cells, we generated an inducible vector to engineer over-expression of wild-type CD38 or an NAD^+^ hydrolase-deficient point mutant [60] of CD38 (E226Q). We introduced wild-type or mutant CD38 into benign RWPE1 cells. Addition of 20 ng/ml doxycycline (Dox) was sufficient to induce CD38 expression within 48 h by western blot (Fig. 4a). Higher levels of Dox caused a significant reduction in intracellular NAD^+^ levels in non-transduced cell lines (Additional file 4) and have been associated with metabolic changes [61], so we chose to carry out our studies using 20 ng/ml Dox. The alamarBlue assay is often used to measure cell viability and proliferation in culture [62]. However, the reagent works as a redox indicator and altered NAD^+^ and NADH levels could influence colorimetric changes used to quantify cell number. As an alternative to alamarBlue, we used DNA quantification to measure relative cell number in order to evaluate cell proliferation over 4 days in culture. With both approaches, we found no significant difference in cell proliferation following expression of wild-type or mutant CD38 (Fig. 4b, Additional file 5a). We utilized an NAD^+^/NADH cycling assay to measure changes in intracellular NAD^+^ and NADH levels upon expression of CD38 relative to total cellular protein. Surprisingly, addition of Dox was not sufficient to alter intracellular NAD^+^, NADH, or NAD^+^:NADH ratio (Fig. 4c–e). Results were replicated using LNCaP and DU145 prostate cancer cells expressing wild-type or mutant CD38 (Additional file 5b–e, Additional file 6a, b). CD38-expressing cells did not show an upregulation of NAMPT or NAPRT, two enzymes that can regenerate NAD^+^ from precursors (Additional file 7a, b). Cells were treated with Triton-X100 to permeabilize cell membranes for 15 min prior to NAD^+^/NADH measurements. Following permeabilization, cells expressing wild-type CD38, but not mutant CD38, demonstrated a dramatic reduction in NAD^+^ levels (Fig. 4f, Additional file 6: Figure S6c,d). These findings suggest that wild-type CD38 exhibits NAD^+^ hydrolase activity in permeabilized but not intact cells. Taken together, we find a lack of evidence to support CD38 as a significant regulator of intracellular NAD^+^ levels or cell proliferation during short-term culture. Considerable depletion of intracellular NAD^+^ and NADH levels by FK866, a small molecule inhibitor of NAMPT, was sufficient to impair cell proliferation of RWPE1, LNCaP, DU145, and PC-3 cells (Fig. 4g–i, Additional file 8a–i), similar to what has been reported for LNCaP and PC-3 cells [32].

### CD38 expression promotes depletion of extracellular NAD^+^

To evaluate extracellular NAD^+^ hydrolase activity, we exposed WT or mutant CD38-expressing cells to exogenous NAD^+^ in culture. Following 30 min, we collected both media and cell extracts and measured the remaining NAD^+^ levels inside and outside of cells. Dox-induced expression of wild-type CD38 significantly reduced extracellular NAD^+^ levels, while no significant differences were observed in cells expressing mutant CD38 (Fig. 4j, Additional file 6e, f). Intracellular levels of NAD^+^ did not increase after adding exogenous NAD^+^ to the media (Additional file 9a–c). We measured a significant net increase in extracellular NAD^+^ over 30 min in culture from naïve LNCaP cells (Additional file 9d) indicating release of intracellular NAD^+^ into the media.

**Fig. 5:**
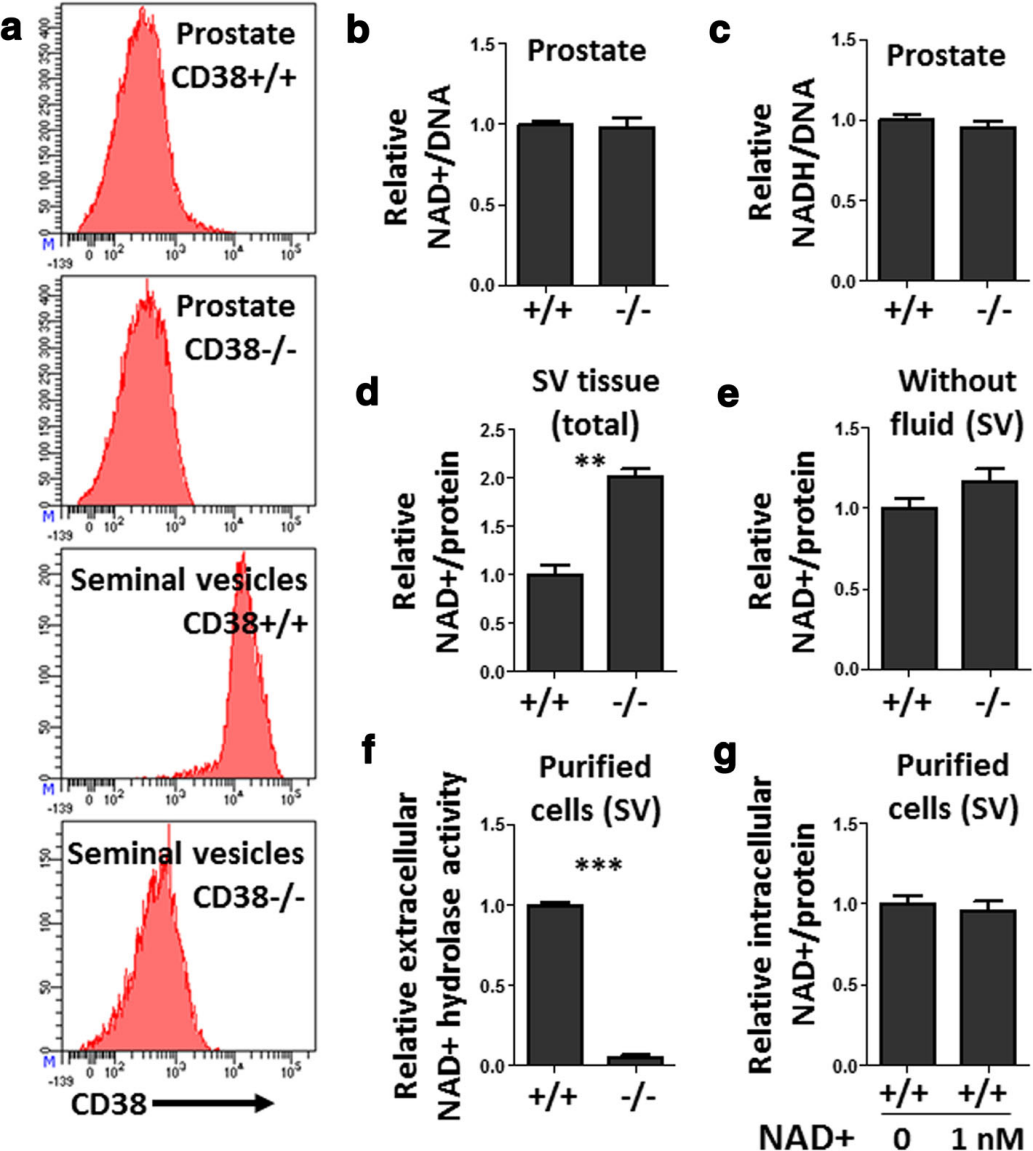
CD38 regulates extracellular NAD^+^ levels in mouse tissues. **a** Flow cytometry histogram plots gated on EpCAM+ epithelial cells from wild-type (CD38+/+) or knockout (CD38−/−) prostate and seminal vesicle cells and stained for surface expression of CD38. **b**, **c** NAD^+^ and NADH levels were measured using the NAD^+^/NADH-Glo assay normalized to total DNA in each tissue and presented relative to wild-type. Mean ± SEM of four replicates is shown. **d**, **e** NAD^+^ levels in intact seminal vesicle tissue (**d**) or in tissue after having removed the fluid (**e**) normalized to protein and presented relative to wild-type. Mean ± SEM of 2–5 replicates is shown. **f** NAD^+^ levels remaining in the media were measured 60 min after the addition of 800 nM exogenous NAD^+^ to purified seminal vesicle cells. Fold reduction in initial NAD^+^ levels, relative to wild-type, is shown. Mean ± SEM of duplicates is shown. **g** Relative intracellular NAD^+^ levels from wild-type seminal vesicle cells measured 60 min after the addition of 0 or 800 nM NAD^+^. Mean ± SEM of three replicates is shown

To extend our findings to an in vivo system, we turned to a mouse model of CD38 deficiency [63]. Consistent with previous reports [64], NAD^+^ levels were elevated in liver tissue from CD38−/− mice compared to wild-type C57BL/6 (CD38+/+) mice (Additional file 10a). We isolated prostate tissue from wild-type and CD38−/− mice (Fig. 5a) and measured NAD^+^ and NADH levels. No significant difference in tissue NAD^+^, NADH levels, or NAD^+^:NADH ratios were observed between wild-type and knockout prostates (Fig. 5b, c, Additional file 10b). The lack of a significant difference in NAD^+^ levels could be due to a low level of CD38 expression in wild-type mouse prostate (Fig. 5a). In contrast to the prostate, seminal vesicle epithelial cells express high levels of CD38 and this expression is abolished in knockout mice (Fig. 5a). Therefore, we reasoned that seminal vesicles may be a better model to assess the role of endogenous CD38 as a regulator of NAD^+^ levels in urogenital tissues. Knockout seminal vesicles exhibited increased levels of NAD^+^ and slightly elevated NAD^+^:NADH ratios compared to CD38-expressing wild-type seminal vesicle tissues (Fig. 5d, Additional file 10c, d). Given that the seminal vesicles contain a large amount of fluid, we removed seminal fluid and measured NAD^+^ levels in the remaining tissue. No significant difference in NAD^+^ levels were observed in wild-type or knockout seminal vesicle tissues after removing the fluid (Fig. 5e), suggesting that external NAD^+^ in fluid is likely to account for the increase in total tissue NAD^+^ levels in knockout seminal vesicles. We measured NAD^+^ levels in the blood of wild-type and knockout mice and found significantly elevated NAD^+^ levels in the plasma of CD38-deficient mice (Additional file 9e). To assess the extracellular NAD^+^ hydrolase activity, we treated purified seminal vesicle cells with exogenous NAD^+^ and evaluated the remaining NAD^+^ in the media after 1 h in culture. Knockout cells exhibited a significant deficiency in depleting extracellular NAD^+^ compared to wild-type seminal vesicle cells (Fig. 5f). The addition of extracellular NAD^+^ to seminal vesicle cells did not cause an increase in intracellular NAD^+^ (Fig. 5g). These findings indicate that CD38 regulates extracellular NAD^+^ levels, with little evidence for regulation of intracellular NAD^+^, in mouse urogenital tissues and human prostate epithelial cell lines.

## Discussion

NAD^+^ is considered an important molecule associated with metabolism, health, and aging, in part through its role as a co-factor for sirtuins and poly-ADP-ribose polymerases (PARPs) [65, 66]. Repletion of NAD^+^ in aging mice upon treatment with precursor molecules like nicotinamide riboside (NR) or nicotinamide mononucleotide (NMN) can reverse age-related tissue failure [29] and reduce the effects of diabetes [67]. NAD^+^ likely plays a critical role in tumorigenesis, as depletion of intracellular NAD^+^ levels by inhibition of NAMPT can impair prostate cancer cell proliferation [32], a result that we reproduced in our study.

CD38 is considered a regulator of intracellular NAD^+^ levels [68]. Pharmacological inhibition of CD38 has been reported to increase intracellular NAD^+^ levels [69]. A previous report in 293T cells showed that CD38 over-expression reduced intracellular NAD^+^ levels by approximately 35% and this was associated with a reduced growth rate in vitro [70]. However, our results using NAMPT inhibitor FK866 indicate that even a 50% reduction in NAD^+^ levels is not sufficient to impair cell proliferation of multiple human prostate cell lines (Fig. 4g–i, Additional file 8). Using mouse models, researchers have demonstrated increased NAD^+^ levels in certain tissues lacking CD38 when compared to wild-type mice [28], which we validated in liver tissue (Additional file 10a). Interestingly, when NAD^+^ levels were measured in various tissues from wild-type or knockout mice, including liver, adipose tissue, spleen, and skeletal muscle, only the liver showed a significant difference in adult mice younger than 32 months of age [64]. We found no difference in NAD^+^ levels when comparing wild-type or CD38 knockout prostates from adult mice, 2–4 months of age. CD38 expression was considerably higher in cells isolated from seminal vesicles than from prostate (Fig. 5a) or liver (Additional file 10f), and we did find elevated NAD^+^ levels in knockout seminal vesicle tissues. However, the trend was not observed after removing fluid, suggesting that elevated NAD^+^ in knockout mice was likely due to differences in the extracellular fluid which make up a considerable portion of seminal vesicle tissues. By combining results from wild-type and CD38 knockout mice, as well as inducible over-expression in cell lines, we find a lack of evidence to support CD38 as a significant regulator of intracellular NAD^+^ in prostate or seminal vesicles.

Several potential explanations may account for differences between published reports and our findings here. For tissues containing a large amount of fluid or blood, measurements of NAD^+^ in whole tissues may be inconsistent with NAD^+^ levels in purified cells from wild-type or CD38 knockout mice. Given that CD38 can rapidly deplete NAD^+^ levels upon permeabilization, any attempts to extract NAD^+^ from cells or tissues without immediately inactivating the CD38 enzymatic activity could lead to reductions in NAD^+^ that are not reflective of NAD^+^ levels in intact cells. Additionally, concentrations of doxycycline used to induce expression of CD38 can influence NAD^+^ levels, as we found statistically significant reductions in intracellular NAD^+^ levels simply by adding 40 or 80 ng/ml doxycycline to naïve RWPE1 and LNCaP cells (Additional file 4). Finally, CD38 may play a fundamentally different role in prostate and seminal vesicles than in the liver. It is not clear if increased NAD^+^ levels in CD38 knockout liver tissues are due to the loss of intracellular or extracellular NAD^+^ hydrolase activity. Given the amount of blood flowing through the liver, and the elevated NAD^+^ levels in the blood of CD38 knockout mice, excess available NAD^+^ may be taken up by knockout liver cells.

We find evidence to support CD38 as a regulator of extracellular NAD^+^, suggesting that repression of CD38 expression in prostate cancer may serve to increase the pool of extracellular NAD^+^. NAD^+^ released from neurons can act as an inhibitory neurotransmitter in gastrointestinal muscles through P2Y receptors in multiple species [71, 72]. Prostate cancer cells release NAD^+^ into the media (Additional file 9d), suggesting that NAD^+^ may serve as an autocrine or paracrine signaling molecule for nearby epithelial or non-epithelial cell types in tumors. CD38 expression and extracellular NAD^+^ hydrolase activity, or the absence of CD38 expression, might influence immune cell proliferation, activation, and survival. NAD^+^ has been shown to inhibit both cytotoxic T cells [33] and regulatory T cells [36], indicating that the make-up of the immune environment may dictate potential immune-suppressing or immune-promoting effects of increased extracellular NAD^+^. The relationship between prostate epithelial expression of CD38 and NAD^+^-induced cell death has not been established. Additionally, a potential role of extracellular NAD^+^ as a signaling molecule in vivo has yet to be investigated in prostate cancer. Further studies will be necessary to determine the effect of extracellular NAD^+^ in the benign and tumor microenvironment. Outside of a functional role in prostate tumorigenesis, we and others have established CD38 as a marker of differentiated prostate luminal cells [7, 56], suggesting that loss of CD38 in prostate cancer may primarily serve as a marker of a de-differentiated or progenitor-like state.

## Conclusions

Reduced expression of CD38 is common in prostate cancer and associated with recurrence after prostatectomy for clinically localized disease, but the mechanisms repressing CD38 expression are poorly understood. In this study, we identify CpG methylation in the *CD38* locus in primary and metastatic prostate cancer. Using inducible expression models in human prostate epithelial cell lines, we found that CD38 over-expression was not sufficient to impair cell proliferation in vitro or alter intracellular NAD^+^ or NADH levels. Using a mouse model of CD38 deficiency, we did not find significant differences in intracellular NAD^+^ levels between wild-type and CD38 knockout prostate or seminal vesicle tissues. However, we found that CD38 expression was sufficient to alter the levels of extracellular NAD^+^, both in human cell lines and primary mouse cells. We therefore propose a model whereby repression of CD38 due to methylation enhances the extracellular pool of NAD^+^ in prostate cancer.

## Additional files

Additional file 1: CpG hypermethylation of *CD38* in prostate cancer cell lines. Related to Fig. 3, methylation heat maps derived from COMPARE-MS analysis of prostate cancer cell lines, referring to the PCR amplicon shown in Fig. 3a (heat map: red—dense methylation; white—no methylation). Note that male white blood cell DNA (WBC) and white blood cell DNA which was in vitro methylated by CpG Methyltransferase M.SssI (WBC SSSI) were used as negative and positive controls, respectively. (TIF 72 kb)

Additional file 2: In silico analysis of TCGA data correlating DNA methylation and mRNA expression of commonly methylated genes in prostate cancer. Correlation plots of log2 mRNA expression (based on RNA-seq, RSEM z-scores) and methylation levels (based on Infinium Human Methylation 450k BeadChip analysis) in 333 primary prostate cancer samples for *GSTP1* and *PTGS2. (TIF 122 kb)*

Additional file 3: Re-expression of *CD38* in prostate cancer cell lines after treatment with DNA methyltransferase inhibitor. Prostate cancer cell lines CWR22rv1, DU145, LNCaP, and LAPC4 were treated with 100 nM, 500 nM 5-aza-2′-deoxycytidine (decitabine, DAC), or solvent (DMSO) for 4 days. Expression of *CD38* was determined by quantitative real-time PCR. Note that a modest re-expression of *CD38* was observed in two (CWR22rv1, LAPC4) out of four cell lines. (TIF 124 kb)

Additional file 4: Effect of Dox on intracellular NAD^+^ levels in RWPE1 and LNCaP cells. (a, b) Intracellular NAD^+^ levels were measured relative to DNA measurements (a) or total cellular protein (b) in naïve RWPE1 (a) and LNCaP (b) cells exposed to varying concentrations of Dox. Results are presented relative to no Dox control. Plot shows mean of 4 replicates per time point ±SEM. Newman-Keuls Multiple Comparison Test. (TIF 67 kb)

Additional file 5: Effect of CD38 expression on RWPE1, LNCaP and DU145 cell proliferation. (a) RWPE1 cell proliferation evaluated using the alamarBlue reagent and measured based on relative absorbance. 0 or 20 ng/mL Dox was used over 4 days. Plots show mean of 3–6 replicates per time point ±SEM. (b, c) Western blot of LNCaP (b) and DU145 (c) cells expressing inducible wild-type or mutant (E226Q) CD38 with or without 20 ng/mL Dox. Tubulin is used as a loading control. (d, e) Cell proliferation evaluated using DNA measurements in LNCaP (d) and DU145 (e) cells. Plots show mean of 5 replicates per time point ± SEM. (TIF 107 kb)

Additional file 6: Effect of CD38 on intracellular/extracellular NAD^+^ levels in LNCaP, DU145 cells. (a, b) NAD^+^ levels were measured relative to total protein in LNCaP (a) and DU145 (b) cells expressing wild-type or mutant CD38 in the presence of 0 or 20 ng/mL Dox presented relative to no Dox (non-induced) sample. Mean ± SEM of 4 replicates is shown. (c, d) LNCaP (c) and DU145 (d) Cells were treated with Triton X-100 (TX-100) to permeabilize cells followed by NAD^+^ measurements. NAD^+^/protein is shown relative to no Dox. Mean ± SEM of 4 replicates is shown. (e, f) Relative NAD^+^/protein levels in the media 30 min after the addition of 800 nM exogenous NAD^+^ to LNCaP (e) and DU145 (f) cells. Mean ± SEM of 4 replicates is shown. (TIF 124 kb)

Additional file 7: Effect of CD38 on expression of enzymes involved in NAD^+^ metabolism. (a, b) Western blots show expression of NAMPT, NAPRT and Tubulin (loading control) in Dox-induced wild-type CD38-expressing RWPE1 (a) and LNCaP (b) cells. (TIF 102 kb)

Additional file 8: NAMPT inhibitor FK866 depletes NAD^+^ levels and impairs proliferation. (a, b, d, e, g, h) Intracellular NAD^+^ and NADH levels were measured in the presence of the indicated concentrations of FK866 in LNCaP (a, b), DU145 (d, e) and PC3 (g, h) cells. Mean ± SEM of 4 replicates is shown. Newman-Keuls Multiple Comparison Test. (c, f, i) Cell proliferation assay over 4 days in culture in the presence of the indicated concentrations of FK866 in LNCaP (c), DU145 (f) and PC3 (i) cells. DNA fluorescence represents relative cell number. 3–6 replicate wells per group per time point were measured. Mean ± SEM is shown. (TIF 131 kb)

Additional file 9: Effect of extracellular NAD^+^ on intracellular NAD^+^ and NADH levels. (a, b) After the addition of exogenous NAD^+^ to the media for 30 min, intracellular NAD^+^ (a) and NADH (b) levels were measured in RWPE1 cells expressing wild-type or mutant CD38. Results are presented as NAD^+^ or NADH relative to protein levels. 20 ng/mL Dox is presented in relation to no Dox (non-induced) samples. Mean ± SEM of 3 replicates is shown. (c) NAD^+^:NADH ratio is calculated based on results shown in A and B. (d) Extracellular NAD^+^ levels (normalized to total protein in the media) were measured using the NAD^+^/NADH-Glo assay 30 min after the addition of fresh media containing 800 nM exogenous NAD^+^ to naïve LNCaP cells. Mean ± SEM of 3 replicates is shown in the presence or absence of Dox. (TIF 94 kb)

Additional file 10: NAD^+^ and NADH levels in wild-type and CD38 knockout mouse tissues. NAD^+^ and NADH levels were measured using the NAD^+^/NADH-Glo assay normalized to total DNA or protein in each tissue and presented relative to wild-type. (a) NAD^+^/protein in livers of knockout compared to wild-type mice. (b) NAD^+^:NADH ratio for prostate tissue is calculated based on results shown in Fig. 5b, c. (c) NADH levels for seminal vesicle tissue. (d) NAD^+^:NADH ratio for seminal vesicle tissue is calculated based on results shown in Fig. 5d and Additional file 10c. Mean ± SEM of 2–4 replicates is shown. (e) NAD^+^ levels were measured in equivalent volumes of plasma isolated from blood of 5 wild-type and 4 knockout mice. (f) Protein was isolated from purified liver or seminal vesicle cells obtained from wild-type adult male mice and probed with antibodies against CD38 or alpha-tubulin by western blot. (TIF 83 kb)

## References

[1] Siegel RL, Miller KD, Jemal A (2018). Cancer statistics, 2018. CA Cancer J Clin.

[2] Scher HI, Sawyers CL (2005). Biology of progressive, castration-resistant prostate cancer: directed therapies targeting the androgen-receptor signaling axis. JClinOncol.

[3] Miao L, Yang L, Li R, Rodrigues DN, Crespo M, Hsieh JT, Tilley WD, de Bono J, Selth LA, Raj GV (2017). Disrupting androgen receptor signaling induces snail-mediated epithelial-mesenchymal plasticity in prostate cancer. Cancer Res.

[4] Ware KE, Somarelli JA, Schaeffer D, Li J, Zhang T, Park S, Patierno SR, Freedman J, Foo WC, Garcia-Blanco MA, Armstrong AJ (2016). Snail promotes resistance to enzalutamide through regulation of androgen receptor activity in prostate cancer. Oncotarget.

[5] Smith BA, Sokolov A, Uzunangelov V, Baertsch R, Newton Y, Graim K, Mathis C, Cheng D, Stuart JM, Witte ON (2015). A basal stem cell signature identifies aggressive prostate cancer phenotypes. Proc Natl Acad Sci U S A.

[6] Markert EK, Mizuno H, Vazquez A, Levine AJ (2011). Molecular classification of prostate cancer using curated expression signatures. Proc Natl Acad Sci U S A.

[7] Liu X, Grogan TR, Hieronymus H, Hashimoto T, Mottahedeh J, Cheng D, Zhang L, Huang K, Stoyanova T, Park JW, Shkhyan RO, Nowroozizadeh B, Rettig MB, Sawyers CL, Elashoff D, Horvath S, Huang J, Witte ON, Goldstein AS (2016). Low CD38 identifies progenitor-like inflammation-associated luminal cells that can initiate human prostate cancer and predict poor outcome. Cell Rep.

[8] Zhang D, Jeter C, Gong S, Tracz A, Lu Y, Shen J, Tang DG. Histone 2B-GFP label-retaining prostate luminal cells possess progenitor cell properties and are intrinsically resistant to castration. Stem Cell Rep. 2018;10(1):228–42. 10.1016/j.stemcr.2017.11.016 PMC5768933 29276153

[9] Qin J, Liu X, Laffin B, Chen X, Choy G, Jeter CR, Calhoun-Davis T, Li H, Palapattu GS, Pang S, Lin K, Huang J, Ivanov I, Li W, Suraneni MV, Tang DG (2012). The PSA(−/lo) prostate cancer cell population harbors self-renewing long-term tumor-propagating cells that resist castration. Cell Stem Cell.

[10] Domingo-Domenech J, Vidal SJ, Rodriguez-Bravo V, Castillo-Martin M, Quinn SA, Rodriguez-Barrueco R, Bonal DM, Charytonowicz E, Gladoun N, de la Iglesia-Vicente J, Petrylak DP, Benson MC, Silva JM, Cordon-Cardo C. Suppression of acquired docetaxel resistance in prostate cancer through depletion of notch- and hedgehog-dependent tumor-initiating cells. Cancer Cell 2012;22(3):373–388. 10.1016/j.ccr.2012.07.016 PMC5989708 22975379

[11] Goldstein AS, Huang J, Guo C, Garraway IP, Witte ON (2010). Identification of a cell of origin for human prostate cancer. Science.

[12] Goldstein AS, Lawson DA, Cheng D, Sun W, Garraway IP, Witte ON (2008). Trop2 identifies a subpopulation of murine and human prostate basal cells with stem cell characteristics. Proc Natl Acad Sci U S A.

[13] Goldstein AS, Drake JM, Burnes DL, Finley DS, Zhang H, Reiter RE, Huang J, Witte ON (2011). Purification and direct transformation of epithelial progenitor cells from primary human prostate. Nat Protoc.

[14] Lukacs RU, Goldstein AS, Lawson DA, Cheng D, Witte ON (2010). Isolation, cultivation and characterization of adult murine prostate stem cells. Nat Protoc.

[15] Karthaus WR, Iaquinta PJ, Drost J, Gracanin A, van Boxtel R, Wongvipat J, Dowling CM, Gao D, Begthel H, Sachs N, Vries RGJ, Cuppen E, Chen Y, Sawyers CL, Clevers HC (2014). Identification of multipotent luminal progenitor cells in human prostate organoid cultures. Cell.

[16] Howard M, Grimaldi JC, Bazan JF, Lund FE, Santos-Argumedo L, Parkhouse RM, Walseth TF, Lee HC (1993). Formation and hydrolysis of cyclic ADP-ribose catalyzed by lymphocyte antigen CD38. Science.

[17] Zocchi E, Franco L, Guida L, Benatti U, Bargellesi A, Malavasi F, Lee HC, De Flora A (1993). A single protein immunologically identified as CD38 displays NAD+ glycohydrolase, ADP-ribosyl cyclase and cyclic ADP-ribose hydrolase activities at the outer surface of human erythrocytes. Biochem Biophys Res Commun.

[18] Adebanjo OA, Anandatheerthavarada HK, Koval AP, Moonga BS, Biswas G, Sun L, Sodam BR, Bevis PJ, Huang CL, Epstein S, Lai FA, Avadhani NG, Zaidi M (1999). A new function for CD38/ADP-ribosyl cyclase in nuclear Ca2+ homeostasis. Nat Cell Biol.

[19] Shrimp JH, Hu J, Dong M, Wang BS, MacDonald R, Jiang H, Hao Q, Yen A, Lin H (2014). Revealing CD38 cellular localization using a cell permeable, mechanism-based fluorescent small-molecule probe. J Am Chem Soc.

[20] Deckert J, Wetzel MC, Bartle LM, Skaletskaya A, Goldmacher VS, Vallee F, Zhou-Liu Q, Ferrari P, Pouzieux S, Lahoute C, Dumontet C, Plesa A, Chiron M, Lejeune P, Chittenden T, Park PU, Blanc V (2014). SAR650984, a novel humanized CD38-targeting antibody, demonstrates potent antitumor activity in models of multiple myeloma and other CD38+ hematologic malignancies. Clin Cancer Res.

[21] Hurtado AM, Chen-Liang TH, Przychodzen B, Hamedi C, Munoz-Ballester J, Dienes B, Garcia-Malo MD, Anton AI, de Arriba F, Teruel-Montoya R, Ortuno FJ, Vicente V, Maciejewski JP, Jerez A (2015). Prognostic signature and clonality pattern of recurrently mutated genes in inactive chronic lymphocytic leukemia. Blood Cancer J.

[22] Lokhorst HM, Plesner T, Laubach JP, Nahi H, Gimsing P, Hansson M, Minnema MC, Lassen U, Krejcik J, Palumbo A, van de Donk NW, Ahmadi T, Khan I, Uhlar CM, Wang J, Sasser AK, Losic N, Lisby S, Basse L, Brun N, Richardson PG (2015). Targeting CD38 with Daratumumab monotherapy in multiple myeloma. N Engl J Med.

[23] Poret N, Fu Q, Guihard S, Cheok M, Miller K, Zeng G, Quesnel B, Troussard X, Galiegue-Zouitina S, Shelley CS (2015). CD38 in hairy cell leukemia is a marker of poor prognosis and a new target for therapy. Cancer Res.

[24] Boini KM, Xia M, Xiong J, Li C, Payne LP, Li PL (2012). Implication of CD38 gene in podocyte epithelial-to-mesenchymal transition and glomerular sclerosis. J Cell Mol Med.

[25] Kramer G, Steiner G, Fodinger D, Fiebiger E, Rappersberger C, Binder S, Hofbauer J, Marberger M (1995). High expression of a CD38-like molecule in normal prostatic epithelium and its differential loss in benign and malignant disease. J Urol.

[26] Liu AY, Roudier MP, True LD (2004). Heterogeneity in primary and metastatic prostate cancer as defined by cell surface CD profile. Am J Pathol.

[27] Kramer G, Steiner GE, Sokol P, Mallone R, Amann G, Marberger M (2003). Loss of CD38 correlates with simultaneous up-regulation of human leukocyte antigen-DR in benign prostatic glands, but not in fetal or androgen-ablated glands, and is strongly related to gland atrophy. BJU Int.

[28] Aksoy P, White TA, Thompson M, Chini EN (2006). Regulation of intracellular levels of NAD: a novel role for CD38. Biochem Biophys Res Commun.

[29] Zhang H, Ryu D, Wu Y, Gariani K, Wang X, Luan P, D'Amico D, Ropelle ER, Lutolf MP, Aebersold R, Schoonjans K, Menzies KJ, Auwerx J (2016). NAD(+) repletion improves mitochondrial and stem cell function and enhances life span in mice. Science.

[30] Aksoy P, Escande C, White TA, Thompson M, Soares S, Benech JC, Chini EN (2006). Regulation of SIRT 1 mediated NAD dependent deacetylation: a novel role for the multifunctional enzyme CD38. Biochem Biophys Res Commun.

[31] Canto C, Menzies KJ, Auwerx J (2015). NAD(+) metabolism and the control of energy homeostasis: a balancing act between mitochondria and the nucleus. Cell Metab.

[32] Wang B, Hasan MK, Alvarado E, Yuan H, Wu H, Chen WY (2011). NAMPT overexpression in prostate cancer and its contribution to tumor cell survival and stress response. Oncogene.

[33] Wang J, Nemoto E, Kots AY, Kaslow HR, Dennert G (1994). Regulation of cytotoxic T cells by ecto-nicotinamide adenine dinucleotide (NAD) correlates with cell surface GPI-anchored/arginine ADP-ribosyltransferase. J Immunol.

[34] Bortell R, Moss J, McKenna RC, Rigby MR, Niedzwiecki D, Stevens LA, Patton WA, Mordes JP, Greiner DL, Rossini AA (2001). Nicotinamide adenine dinucleotide (NAD) and its metabolites inhibit T lymphocyte proliferation: role of cell surface NAD glycohydrolase and pyrophosphatase activities. J Immunol.

[35] Seman M, Adriouch S, Scheuplein F, Krebs C, Freese D, Glowacki G, Deterre P, Haag F, Koch-Nolte F (2003). NAD-induced T cell death: ADP-ribosylation of cell surface proteins by ART2 activates the cytolytic P2X7 purinoceptor. Immunity.

[36] Hubert S, Rissiek B, Klages K, Huehn J, Sparwasser T, Haag F, Koch-Nolte F, Boyer O, Seman M, Adriouch S (2010). Extracellular NAD+ shapes the Foxp3+ regulatory T cell compartment through the ART2-P2X7 pathway. J Exp Med.

[37] Hamid O, Robert C, Daud A, Hodi FS, Hwu WJ, Kefford R, Wolchok JD, Hersey P, Joseph RW, Weber JS, Dronca R, Gangadhar TC, Patnaik A, Zarour H, Joshua AM, Gergich K, Elassaiss-Schaap J, Algazi A, Mateus C, Boasberg P, Tumeh PC, Chmielowski B, Ebbinghaus SW, Li XN, Kang SP, Ribas A (2013). Safety and tumor responses with lambrolizumab (anti-PD-1) in melanoma. N Engl J Med.

[38] Yegnasubramanian S, Lin X, Haffner MC, DeMarzo AM, Nelson WG (2006). Combination of methylated-DNA precipitation and methylation-sensitive restriction enzymes (COMPARE-MS) for the rapid, sensitive and quantitative detection of DNA methylation. Nucleic Acids Res.

[39] Yegnasubramanian S, Kowalski J, Gonzalgo ML, Zahurak M, Piantadosi S, Walsh PC, Bova GS, De Marzo AM, Isaacs WB, Nelson WG (2004). Hypermethylation of CpG islands in primary and metastatic human prostate cancer. Cancer Res.

[40] Yegnasubramanian S, Wu Z, Haffner MC, Esopi D, Aryee MJ, Badrinath R, He TL, Morgan JD, Carvalho B, Zheng Q, De Marzo AM, Irizarry RA, Nelson WG (2011). Chromosome-wide mapping of DNA methylation patterns in normal and malignant prostate cells reveals pervasive methylation of gene-associated and conserved intergenic sequences. BMC Genomics.

[41] Cancer Genome Atlas Research N (2015). The molecular taxonomy of primary prostate cancer. Cell.

[42] Gao J, Aksoy BA, Dogrusoz U, Dresdner G, Gross B, Sumer SO, Sun Y, Jacobsen A, Sinha R, Larsson E, Cerami E, Sander C, Schultz N (2013). Integrative analysis of complex cancer genomics and clinical profiles using the cBioPortal. Sci Signal.

[43] Beltran H, Rickman DS, Park K, Chae SS, Sboner A, MacDonald TY, Wang Y, Sheikh KL, Terry S, Tagawa ST, Dhir R, Nelson JB, de la Taille A, Allory Y, Gerstein MB, Perner S, Pienta KJ, Chinnaiyan AM, Wang Y, Collins CC, Gleave ME, Demichelis F, Nanus DM, Rubin MA (2011). Molecular characterization of neuroendocrine prostate cancer and identification of new drug targets. Cancer Discov.

[44] Chakravarty D, Sboner A, Nair SS, Giannopoulou E, Li R, Hennig S, Mosquera JM, Pauwels J, Park K, Kossai M, MacDonald TY, Fontugne J, Erho N, Vergara IA, Ghadessi M, Davicioni E, Jenkins RB, Palanisamy N, Chen Z, Nakagawa S, Hirose T, Bander NH, Beltran H, Fox AH, Elemento O, Rubin MA (2014). The oestrogen receptor alpha-regulated lncRNA NEAT1 is a critical modulator of prostate cancer. Nat Commun.

[45] Beltran H, Prandi D, Mosquera JM, Benelli M, Puca L, Cyrta J, Marotz C, Giannopoulou E, Chakravarthi BV, Varambally S, Tomlins SA, Nanus DM, Tagawa ST, Van Allen EM, Elemento O, Sboner A, Garraway LA, Rubin MA, Demichelis F (2016). Divergent clonal evolution of castration-resistant neuroendocrine prostate cancer. Nat Med.

[46] Dobin A, Davis CA, Schlesinger F, Drenkow J, Zaleski C, Jha S, Batut P, Chaisson M, Gingeras TR (2013). STAR: ultrafast universal RNA-seq aligner. Bioinformatics.

[47] Li H, Handsaker B, Wysoker A, Fennell T, Ruan J, Homer N, Marth G, Abecasis G, Durbin R, Genome Project Data Processing S (2009). The sequence alignment/map format and SAMtools. Bioinformatics.

[48] Trapnell C, Roberts A, Goff L, Pertea G, Kim D, Kelley DR, Pimentel H, Salzberg SL, Rinn JL, Pachter L (2012). Differential gene and transcript expression analysis of RNA-seq experiments with TopHat and cufflinks. Nat Protoc.

[49] Derrien T, Johnson R, Bussotti G, Tanzer A, Djebali S, Tilgner H, Guernec G, Martin D, Merkel A, Knowles DG, Lagarde J, Veeravalli L, Ruan X, Ruan Y, Lassmann T, Carninci P, Brown JB, Lipovich L, Gonzalez JM, Thomas M, Davis CA, Shiekhattar R, Gingeras TR, Hubbard TJ, Notredame C, Harrow J, Guigo R (2012). The GENCODE v7 catalog of human long noncoding RNAs: analysis of their gene structure, evolution, and expression. Genome Res.

[50] Leek JT, Johnson WE, Parker HS, Jaffe AE, Storey JD (2012). The sva package for removing batch effects and other unwanted variation in high-throughput experiments. Bioinformatics.

[51] Hempel HA, Cuka NS, Kulac I, Barber JR, Cornish TC, Platz EA, De Marzo AM, Sfanos KS (2017). Low intratumoral mast cells are associated with a higher risk of prostate cancer recurrence. Prostate.

[52] Toubaji A, Albadine R, Meeker AK, Isaacs WB, Lotan T, Haffner MC, Chaux A, Epstein JI, Han M, Walsh PC, Partin AW, De Marzo AM, Platz EA, Netto GJ (2011). Increased gene copy number of ERG on chromosome 21 but not TMPRSS2-ERG fusion predicts outcome in prostatic adenocarcinomas. Mod Pathol.

[53] Breslow NE, Day NE (1980). Statistical methods in cancer research. Volume I - The analysis of case-control studies. IARC Sci Publ.

[54] Gollapudi K, Galet C, Grogan T, Zhang H, Said JW, Huang J, Elashoff D, Freedland SJ, Rettig M, Aronson WJ (2013). Association between tumor-associated macrophage infiltration, high grade prostate cancer, and biochemical recurrence after radical prostatectomy. Am J Cancer Res.

[55] Aryee MJ, Liu W, Engelmann JC, Nuhn P, Gurel M, Haffner MC, Esopi D, Irizarry RA, Getzenberg RH, Nelson WG, Luo J, Xu J, Isaacs WB, Bova GS, Yegnasubramanian S (2013). DNA methylation alterations exhibit intraindividual stability and interindividual heterogeneity in prostate cancer metastases. Sci Transl Med.

[56] Sahoo D, Wei W, Auman H, Hurtado-Coll A, Carroll PR, Fazli L, Gleave ME, Lin DW, Nelson PS, Simko J, Thompson IM, Leach RJ, Troyer DA, True LD, McKenney JK, Feng Z, Brooks JD (2018). Boolean analysis identifies CD38 as a biomarker of aggressive localized prostate cancer. Oncotarget.

[57] Meissner A, Mikkelsen TS, Gu H, Wernig M, Hanna J, Sivachenko A, Zhang X, Bernstein BE, Nusbaum C, Jaffe DB, Gnirke A, Jaenisch R, Lander ES (2008). Genome-scale DNA methylation maps of pluripotent and differentiated cells. Nature.

[58] Yegnasubramanian S, Haffner MC, Zhang Y, Gurel B, Cornish TC, Wu Z, Irizarry RA, Morgan J, Hicks J, DeWeese TL, Isaacs WB, Bova GS, De Marzo AM, Nelson WG (2008). DNA hypomethylation arises later in prostate cancer progression than CpG island hypermethylation and contributes to metastatic tumor heterogeneity. Cancer Res.

[59] Sharad S, Ravindranath L, Haffner MC, Li H, Yan W, Sesterhenn IA, Chen Y, Ali A, Srinivasan A, McLeod DG, Yegnasubramanian S, Srivastava S, Dobi A, Petrovics G (2014). Methylation of the PMEPA1 gene, a negative regulator of the androgen receptor in prostate cancer. Epigenetics.

[60] Congleton J, Jiang H, Malavasi F, Lin H, Yen A (2011). ATRA-induced HL-60 myeloid leukemia cell differentiation depends on the CD38 cytosolic tail needed for membrane localization, but CD38 enzymatic activity is unnecessary. Exp Cell Res.

[61] Ahler E, Sullivan WJ, Cass A, Braas D, York AG, Bensinger SJ, Graeber TG, Christofk HR (2013). Doxycycline alters metabolism and proliferation of human cell lines. PLoS One.

[62] Page B, Page M, Noel C (1993). A new fluorometric assay for cytotoxicity measurements in-vitro. Int J Oncol.

[63] Cockayne DA, Muchamuel T, Grimaldi JC, Muller-Steffner H, Randall TD, Lund FE, Murray R, Schuber F, Howard MC (1998). Mice deficient for the ecto-nicotinamide adenine dinucleotide glycohydrolase CD38 exhibit altered humoral immune responses. Blood.

[64] Camacho-Pereira J, Tarrago MG, Chini CCS, Nin V, Escande C, Warner GM, Puranik AS, Schoon RA, Reid JM, Galina A, Chini EN (2016). CD38 dictates age-related NAD decline and mitochondrial dysfunction through an SIRT3-dependent mechanism. Cell Metab.

[65] Yoshino J, Baur JA, Imai SI (2018). NAD(+) intermediates: the biology and therapeutic potential of NMN and NR. Cell Metab.

[66] Rajman L, Chwalek K, Sinclair DA (2018). Therapeutic potential of NAD-boosting molecules: the in vivo evidence. Cell Metab.

[67] Yoshino J, Mills KF, Yoon MJ, Imai S (2011). Nicotinamide mononucleotide, a key NAD(+) intermediate, treats the pathophysiology of diet- and age-induced diabetes in mice. Cell Metab.

[68] Chini EN (2009). CD38 as a regulator of cellular NAD: a novel potential pharmacological target for metabolic conditions. Curr Pharm Des.

[69] Escande C, Nin V, Price NL, Capellini V, Gomes AP, Barbosa MT, O'Neil L, White TA, Sinclair DA, Chini EN (2013). Flavonoid apigenin is an inhibitor of the NAD+ ase CD38: implications for cellular NAD+ metabolism, protein acetylation, and treatment of metabolic syndrome. Diabetes.

[70] Hu Y, Wang H, Wang Q, Deng H (2014). Overexpression of CD38 decreases cellular NAD levels and alters the expression of proteins involved in energy metabolism and antioxidant defense. J Proteome Res.

[71] Mutafova-Yambolieva VN, Hwang SJ, Hao X, Chen H, Zhu MX, Wood JD, Ward SM, Sanders KM (2007). Beta-nicotinamide adenine dinucleotide is an inhibitory neurotransmitter in visceral smooth muscle. Proc Natl Acad Sci U S A.

[72] Hwang SJ, Durnin L, Dwyer L, Rhee PL, Ward SM, Koh SD, Sanders KM, Mutafova-Yambolieva VN (2011). Beta-nicotinamide adenine dinucleotide is an enteric inhibitory neurotransmitter in human and nonhuman primate colons. Gastroenterology.

