# Supplementary figures and images for: CD38 is methylated in prostate cancer and regulates extracellular NAD+
Source: Cancer Metab. 2018 Sep 21;6:13. doi: 10.1186/s40170-018-0186-3 (PMC6150989; doi:10.1186/s40170-018-0186-3)

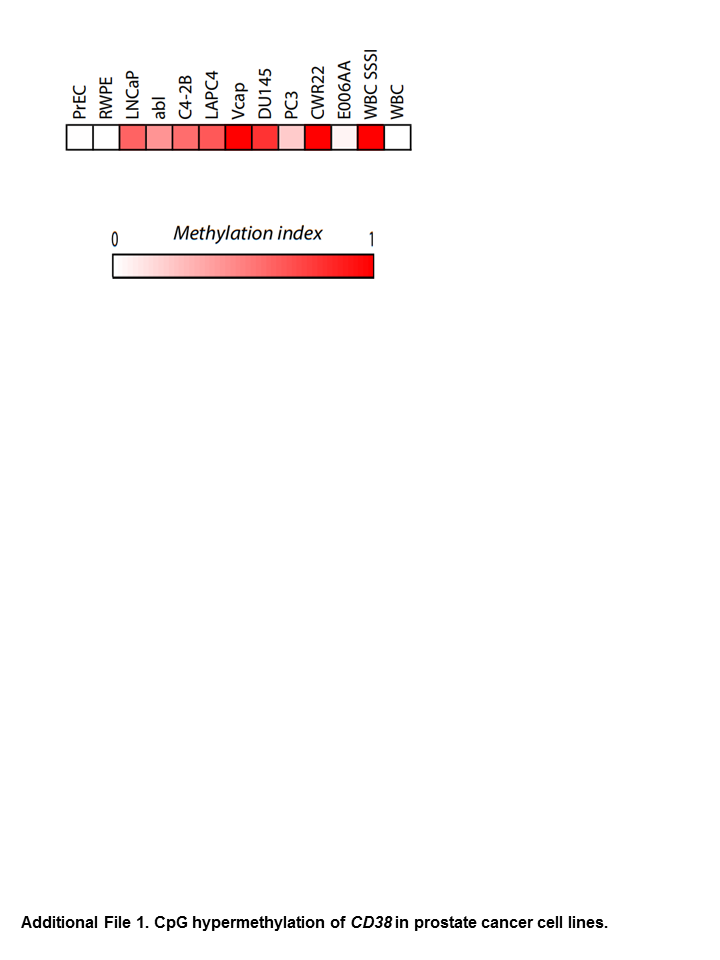

Supplement: Supplementary file 1 — CpG hypermethylation of CD38 in prostate cancer cell lines. Related to Fig. 3, methylation heat maps derived from COMPARE-MS analysis of prostate cancer cell lines, referring to the PCR amplicon shown in Fig. 3a (heat map: red—dense methylation; white—no methylation). Note that male white blood cell DNA (WBC) and white blood cell DNA which was in vitro methylated by CpG Methyltransferase M.SssI (WBC SSSI) were used as negative and positive controls, respectively. (TIF 72 kb) [file 40170_2018_186_MOESM1_ESM.tif]

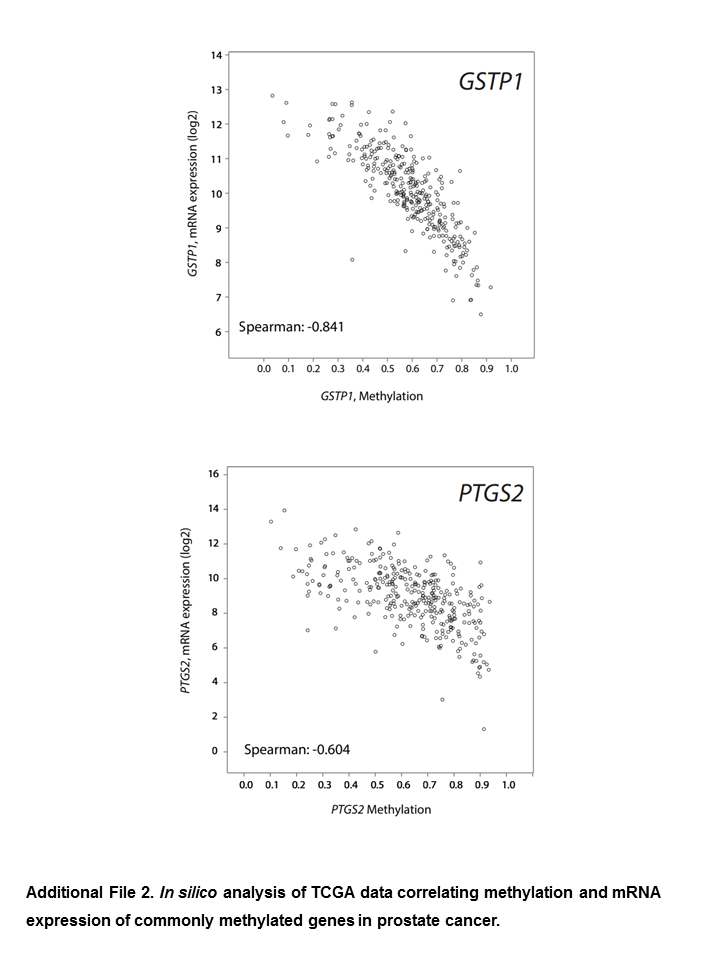

Supplement: Supplementary file 2 — In silico analysis of TCGA data correlating DNA methylation and mRNA expression of commonly methylated genes in prostate cancer. Correlation plots of log2 mRNA expression (based on RNA-seq, RSEM z-scores) and methylation levels (based on Infinium Human Methylation 450k BeadChip analysis) in 333 primary prostate cancer samples for GSTP1 and PTGS2. (TIF 122 kb) [file 40170_2018_186_MOESM2_ESM.tif]

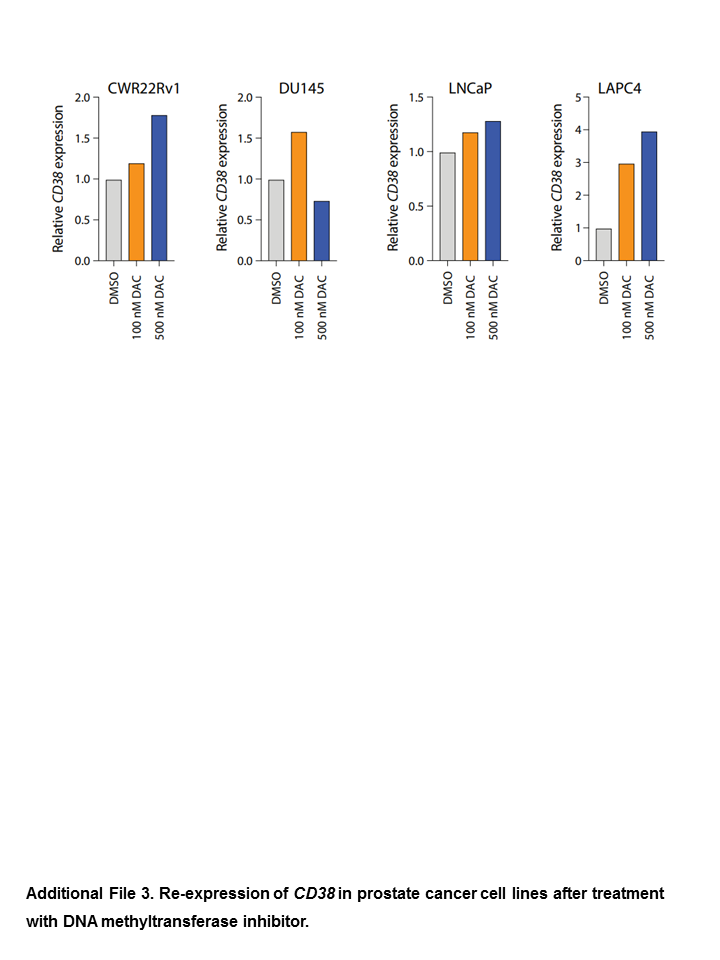

Supplement: Supplementary file 3 — Re-expression of CD38 in prostate cancer cell lines after treatment with DNA methyltransferase inhibitor. Prostate cancer cell lines CWR22rv1, DU145, LNCaP, and LAPC4 were treated with 100 nM, 500 nM 5-aza-2′-deoxycytidine (decitabine, DAC), or solvent (DMSO) for 4 days. Expression of CD38 was determined by quantitative real-time PCR. Note that a modest re-expression of CD38 was observed in two (CWR22rv1, LAPC4) out of four cell lines. (TIF 124 kb) [file 40170_2018_186_MOESM3_ESM.tif]

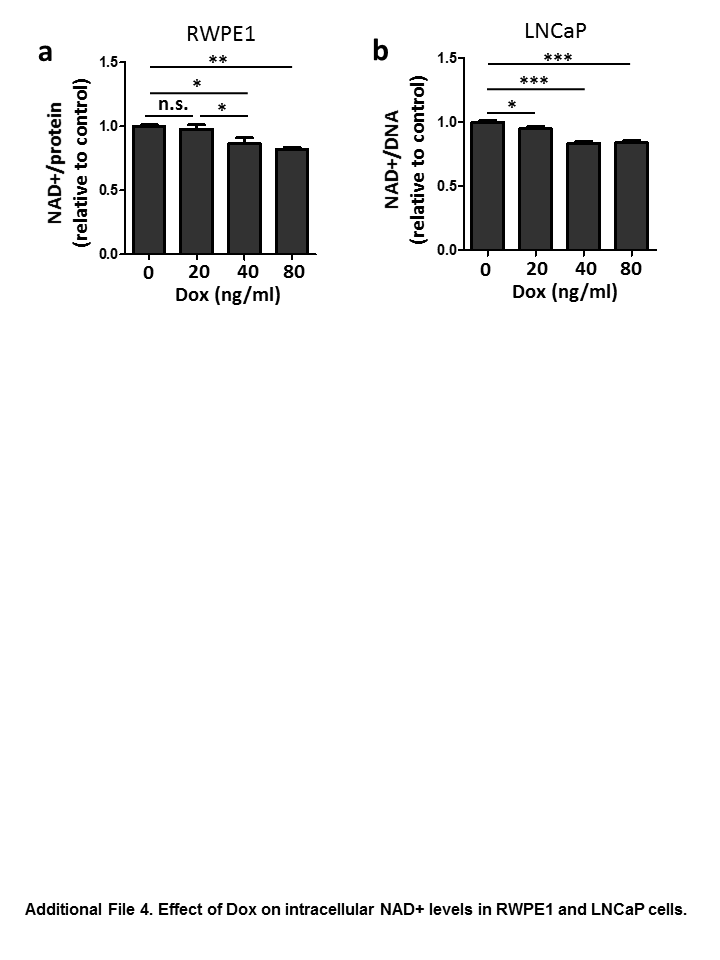

Supplement: Supplementary file 4 — Effect of Dox on intracellular NAD+ levels in RWPE1 and LNCaP cells. (a, b) Intracellular NAD+ levels were measured relative to DNA measurements (a) or total cellular protein (b) in naïve RWPE1 (a) and LNCaP (b) cells exposed to varying concentrations of Dox. Results are presented relative to no Dox control. Plot shows mean of 4 replicates per time point ±SEM. Newman-Keuls Multiple Comparison Test. (TIF 67 kb) [file 40170_2018_186_MOESM4_ESM.tif]

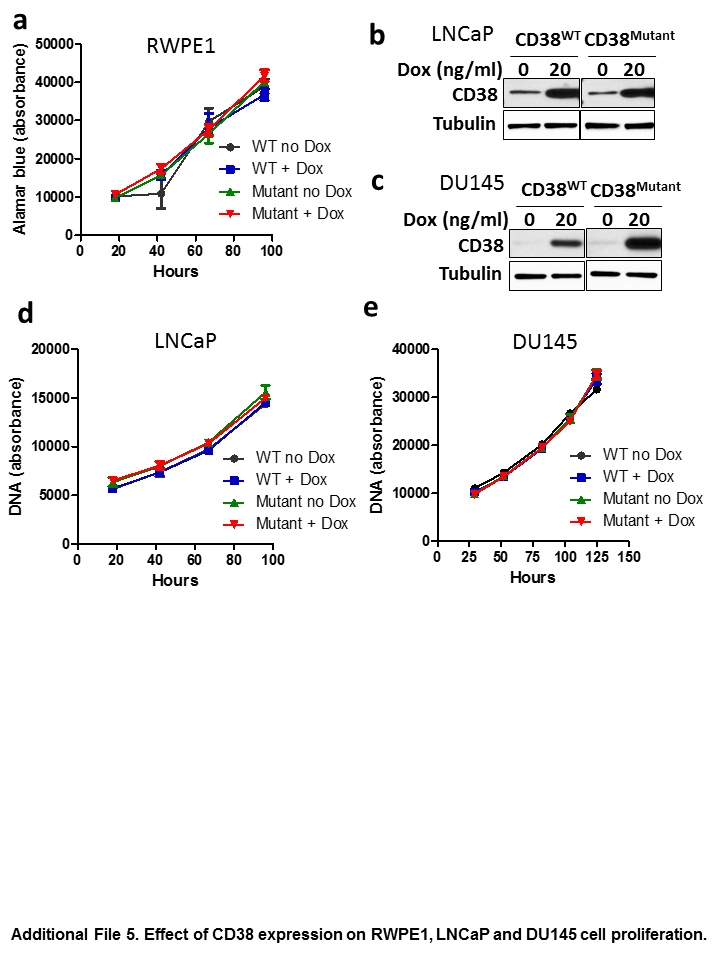

Supplement: Supplementary file 5 — Effect of CD38 expression on RWPE1, LNCaP and DU145 cell proliferation. (a) RWPE1 cell proliferation evaluated using the alamarBlue reagent and measured based on relative absorbance. 0 or 20 ng/mL Dox was used over 4 days. Plots show mean of 3–6 replicates per time point ±SEM. (b, c) Western blot of LNCaP (b) and DU145 (c) cells expressing inducible wild-type or mutant (E226Q) CD38 with or without 20 ng/mL Dox. Tubulin is used as a loading control. (d, e) Cell proliferation evaluated using DNA measurements in LNCaP (d) and DU145 (e) cells. Plots show mean of 5 replicates per time point ± SEM. (TIF 107 kb) [file 40170_2018_186_MOESM5_ESM.tif]

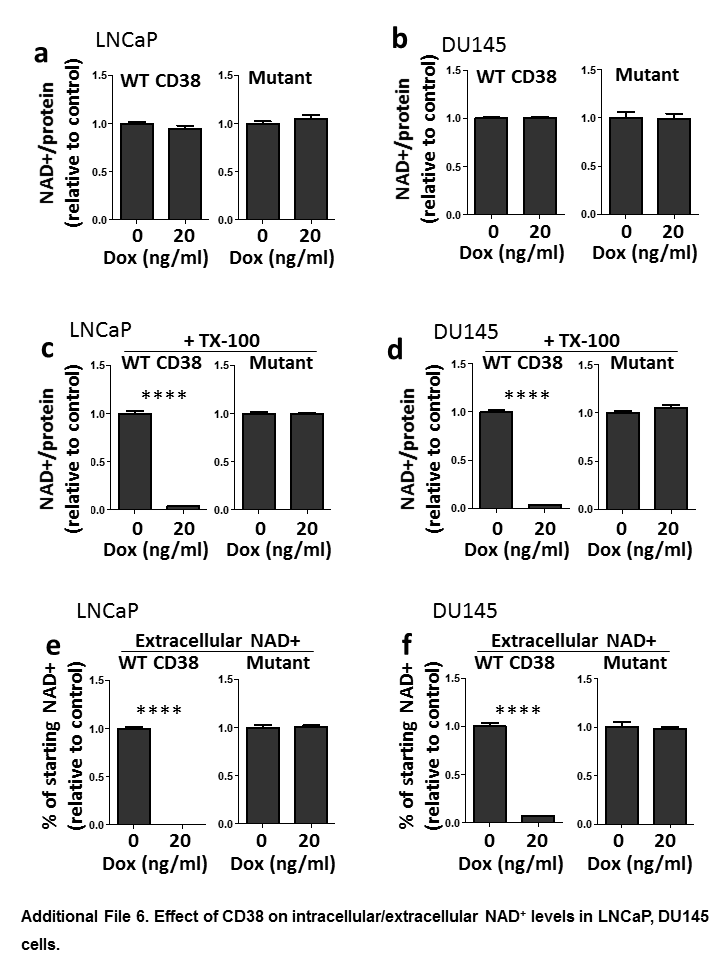

Supplement: Supplementary file 6 — Effect of CD38 on intracellular/extracellular NAD+ levels in LNCaP, DU145 cells. (a, b) NAD+ levels were measured relative to total protein in LNCaP (a) and DU145 (b) cells expressing wild-type or mutant CD38 in the presence of 0 or 20 ng/mL Dox presented relative to no Dox (non-induced) sample. Mean ± SEM of 4 replicates is shown. (c, d) LNCaP (c) and DU145 (d) Cells were treated with Triton X-100 (TX-100) to permeabilize cells followed by NAD+ measurements. NAD+/protein is shown relative to no Dox. Mean ± SEM of 4 replicates is shown. (e, f) Relative NAD+/protein levels in the media 30 min after the addition of 800 nM exogenous NAD+ to LNCaP (e) and DU145 (f) cells. Mean ± SEM of 4 replicates is shown. (TIF 124 kb) [file 40170_2018_186_MOESM6_ESM.tif]

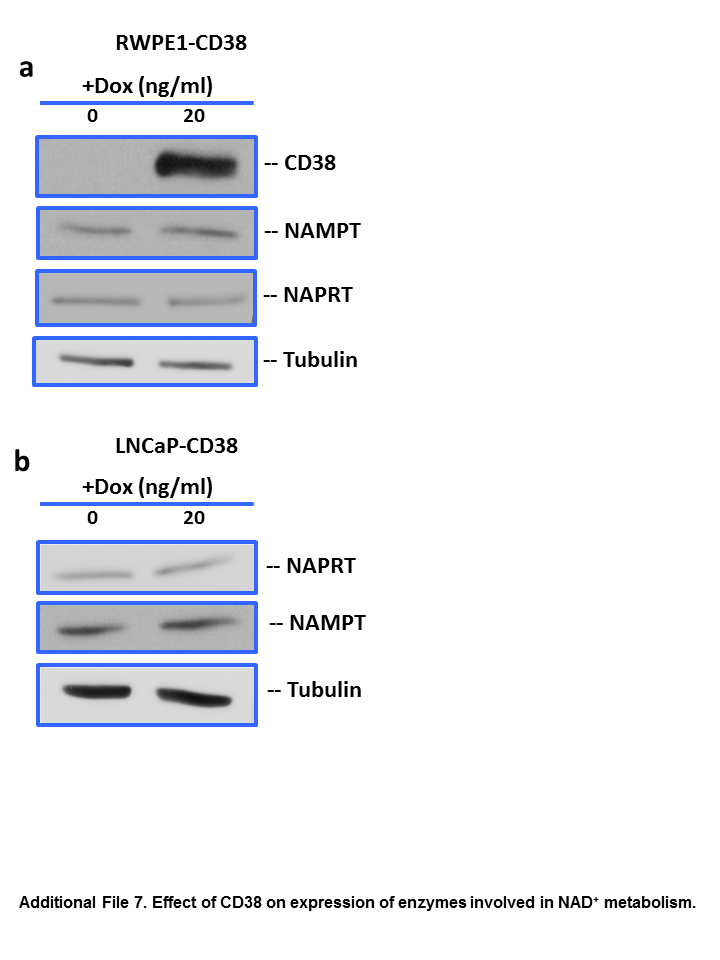

Supplement: Supplementary file 7 — Effect of CD38 on expression of enzymes involved in NAD+ metabolism. (a, b) Western blots show expression of NAMPT, NAPRT and Tubulin (loading control) in Dox-induced wild-type CD38-expressing RWPE1 (a) and LNCaP (b) cells. (TIF 102 kb) [file 40170_2018_186_MOESM7_ESM.tif]

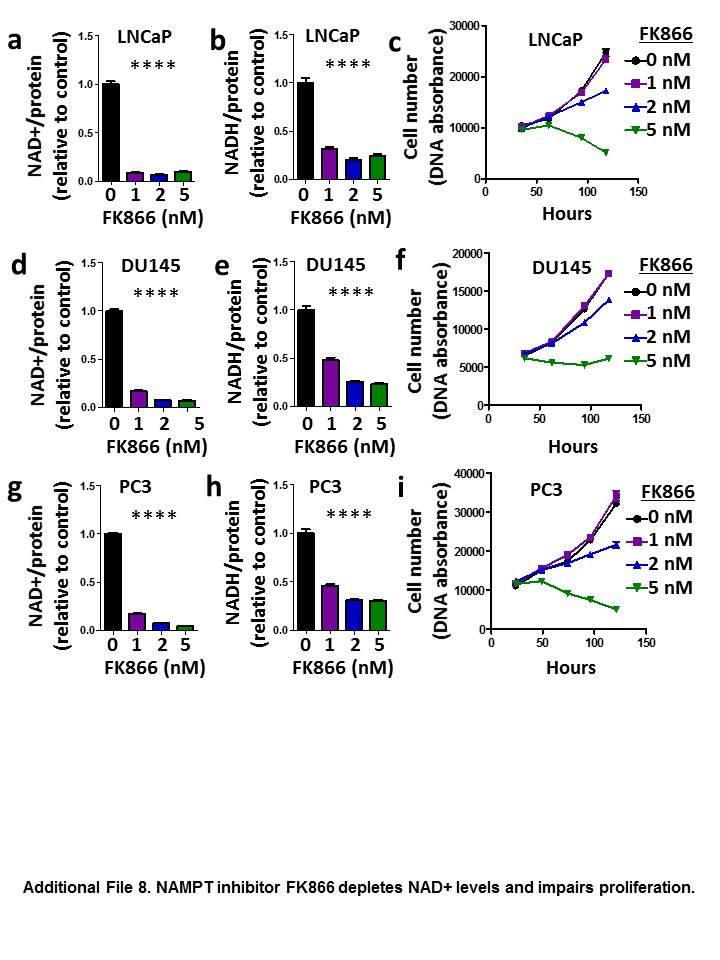

Supplement: Supplementary file 8 — NAMPT inhibitor FK866 depletes NAD+ levels and impairs proliferation. (a, b, d, e, g, h) Intracellular NAD+ and NADH levels were measured in the presence of the indicated concentrations of FK866 in LNCaP (a, b), DU145 (d, e) and PC3 (g, h) cells. Mean ± SEM of 4 replicates is shown. Newman-Keuls Multiple Comparison Test. (c, f, i) Cell proliferation assay over 4 days in culture in the presence of the indicated concentrations of FK866 in LNCaP (c), DU145 (f) and PC3 (i) cells. DNA fluorescence represents relative cell number. 3–6 replicate wells per group per time point were measured. Mean ± SEM is shown. (TIF 131 kb) [file 40170_2018_186_MOESM8_ESM.tif]

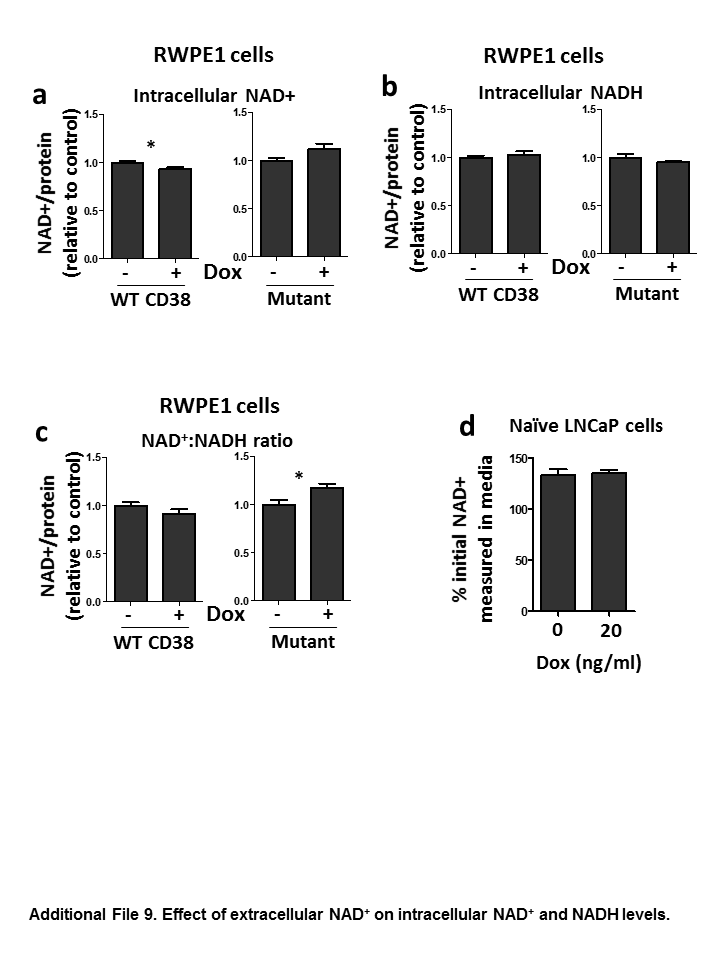

Supplement: Supplementary file 9 — Effect of extracellular NAD+ on intracellular NAD+ and NADH levels. (a, b) After the addition of exogenous NAD+ to the media for 30 min, intracellular NAD+ (a) and NADH (b) levels were measured in RWPE1 cells expressing wild-type or mutant CD38. Results are presented as NAD+ or NADH relative to protein levels. 20 ng/mL Dox is presented in relation to no Dox (non-induced) samples. Mean ± SEM of 3 replicates is shown. (c) NAD+:NADH ratio is calculated based on results shown in A and B. (d) Extracellular NAD+ levels (normalized to total protein in the media) were measured using the NAD+/NADH-Glo assay 30 min after the addition of fresh media containing 800 nM exogenous NAD+ to naïve LNCaP cells. Mean ± SEM of 3 replicates is shown in the presence or absence of Dox. (TIF 94 kb) [file 40170_2018_186_MOESM9_ESM.tif]

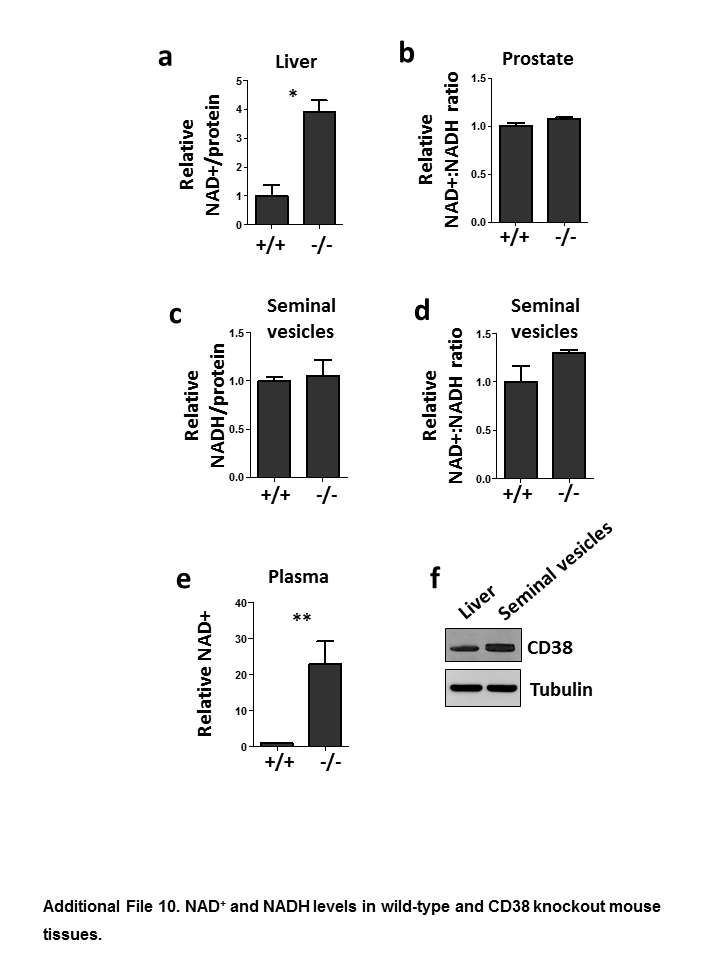

Supplement: Supplementary file 10 — NAD+ and NADH levels in wild-type and CD38 knockout mouse tissues. NAD+ and NADH levels were measured using the NAD+/NADH-Glo assay normalized to total DNA or protein in each tissue and presented relative to wild-type. (a) NAD+/protein in livers of knockout compared to wild-type mice. (b) NAD+:NADH ratio for prostate tissue is calculated based on results shown in Fig. 5b, c. (c) NADH levels for seminal vesicle tissue. (d) NAD+:NADH ratio for seminal vesicle tissue is calculated based on results shown in Fig. 5d and Additional file 10c. Mean ± SEM of 2–4 replicates is shown. (e) NAD+ levels were measured in equivalent volumes of plasma isolated from blood of 5 wild-type and 4 knockout mice. (f) Protein was isolated from purified liver or seminal vesicle cells obtained from wild-type adult male mice and probed with antibodies against CD38 or alpha-tubulin by western blot. (TIF 83 kb) [file 40170_2018_186_MOESM10_ESM.tif]
